# Roadmap for light interaction with biophotonic surfaces and their diverse applications

**DOI:** 10.1117/1.JBO.31.6.064302

**Published:** 2026-02-14

**Authors:** Adam Władziński, Igor Meglinski, Alexander Bykov, Maria Gritsevich, Mikhail Kryuchkov, Vladimir L. Katanaev, Brindusa Dragoi, Nicolina Pop, Junyoung Kwon, Susete N. Fernandes, Maria Helena Godinho, Savvas G. Chalkidis, Georgios C. Vougioukalakis, Atle M. Bones, Maciej S. Wróbel, Katarzyna Karpienko, Marta Władzińska, Patryk Sokołowski, Tatiana Novikova, Jessica C. Ramella-Roman, Jošt Stergar, Urban Simončič, Matija Milanič, Nikola Vuković, Jelena Radovanović, Aleksandar Demić, Dragan Indjin, Marcin Gnyba, Małgorzata Szczerska

**Affiliations:** a Gdańsk University of Technology, Department of Metrology and Optoelectronics, Faculty of Electronics, Telecommunications and Informatics, Gdańsk, Poland; b ECO CHAIN, Gdynia, Poland; c Aston University, College of Engineering and Physical Sciences, Birmingham, United Kingdom; d University of Oulu, Faculty of Information Technology and Electrical Engineering, Oulu, Finland; e University of Helsinki, Faculty of Science, Helsinki, Finland; f Ural Federal University, Institute of Physics and Technology, Ekaterinburg, Russia; g University of Geneva, Faculty of Medicine, Geneva, Switzerland; h Regional Institute of Oncology, TRANSCEND Research Center, Nanotechnology Laboratory, Iasi, Romania; I Al. I. Cuza University, Faculty of Chemistry, Iasi, Romania; j Politehnica University of Timisoara, Department of Physical Foundations of Engineering, Timisoara, Romania; k Pukyong National University, Major of Nanotechnology Engineering, Busan, Republic of Korea; l NOVA University Lisbon, NOVA School of Science and Technology, i3N/CENIMAT, Department of Materials Science, Caparica, Portugal; m National and Kapodistrian University of Athens, Department of Chemistry, Laboratory of Organic Chemistry, Athens, Greece; n Norwegian University of Science and Technology, Department of Biology, Cell, Molecular Biology and Genomics Group, Trondheim, Norway; o IP Paris, Ecole Polytechnique, CNRS, LPICM, Palaiseau, France; p Florida International University, Department of Biomedical Engineering, Miami, Florida, United States; q Florida International University, Department of Ophthalmology, Herbert Wertheim College of Medicine, Miami, Florida, United States; r Jozef Stefan Institute, Ljubljana, Slovenia; s University of Ljubljana, Faculty of Mathematics and Physics, Ljubljana, Slovenia; t University of Belgrade, School of Electrical Engineering, Beograd, Serbia; u University of Leeds, School of Electronic and Electrical Engineering, Leeds, United Kingdom

**Keywords:** bio-inspired structures, biophotonics phantoms, nanostructures, nanosurfaces, light–tissue interaction

## Abstract

**Significance:**

Biophotonics has advanced through many discoveries, yet challenges remain, including label-free biomolecular specificity, quantitative imaging, and single-molecule detection. Progress is further constrained by the need for cheaper, lighter, miniaturized materials that still meet strict optical, electrical, and mechanical specifications. This limitation can be overcome if bioinspired structures are developed. One of the developed areas in which solutions in nature are used is micro and nanostructures including nanosurfaces. It offers a way to increase biomolecular specificity and develop lightweight, low-cost devices for biomedicine. However, it requires measuring phenomena in materials and testing these materials in applications, e.g., sensing systems.

**Aim:**

We offer a concise, authoritative overview of biophotonics—from nanoscale light–biomolecule interactions to bioinspired materials, phantoms, test methods, and sensor development.

**Approach:**

A coherent and comprehensive analysis of the crucial problems related to the development of bioinspired materials and devices was carried out. Recent advances in light scattering by biological surfaces enable structure characterization, disease diagnosis, red-blood-cell analysis, drug discovery, and optical imaging and sensing. Structural and genetic bases of biological photonic surfaces were examined, alongside key performance factors in bio-inspired materials—biocompatibility, biodegradability, structure-optics coupling (e.g., dynamic color change), and scalability limits. We survey chiral nanomaterials, silica frustules, and artificial surfaces that emulate peacock feathers, butterfly wings, iridescent fruits, plant petals, and beetle cuticles, highlighting complementary diagnostics—omics, hyperspectral, and terahertz imaging—for structural analysis and material innovation. We examine bio-inspired phantoms for medical calibration, recent advances in Monte Carlo tissue light-transport modeling, and the resulting applications of these materials and diagnostic tools.

**Results:**

Results confirm a broad set of tunable bio-inspired materials: key optical phenomena were mapped, structures fabricated and modeled, phantoms validated, and strong sensor potential demonstrated.

**Conclusions:**

We survey emerging biophotonics, review material and system requirements, and emphasize simplifying and miniaturizing sensors for biomedical use.

## 1 Introduction

**Section Authors**: Marcin Gnyba (Gdańsk University of Technology) and Adam Władziński (Gdańsk University of Technology)

The earliest known research works in biophotonics were as early as the sixth century BC, and they related to the Greek philosophers from Miletus: Anaximander and Anaximenes, who had the first documented bioluminescence. Similarly, the concepts of developing new technical solutions by imitating solutions existing in nature date back to ancient times. The evolution of biophotonics has been highlighted by numerous discoveries and inventions, including the Nobel Prize for the development of super-resolved fluorescence microscopy in 2014, which enabled the study of living cells at the molecular level and observation of single proteins at a level of detail not seen before. Still, despite long-lasting and intense development, many challenges remain current, e.g., achieving biomolecular specificity label-free, developing quantitative imaging and sensing modalities, and reaching single molecule detection. Moreover, significant limitations to the development of biophotonics are related to the requirements of reducing the costs of materials, miniaturization, and reducing their weight, combined with maintaining the required optical, electrical, and mechanical parameters. This limitation can be overcome if new, bioinspired structures are developed. One of the currently developed areas in which solutions existing in nature are used is photonic micro and nanostructures including nanosurfaces. It offers a powerful way to increase biomolecular specificity and develop new low-weight and low-cost devices for biomedicine. However, it requires not only measuring and using new phenomena in materials but also testing these materials in new applications, e.g., sensing systems.

This roadmap article aims to provide a concise and authoritative overview of the present and future of biophotonics, spanning from the detailed study of light–matter interaction phenomena with biomolecules at the nanoscale through the development of bioinspired photonic materials and surfaces up to the development of biophotonic sensors.

Section 2 discusses recent advances associated with light scattering by biological surfaces having several perspectives due to important applications in various fields of biomedicine and related areas. Some of the key perspectives include characterization of biological structures, diagnosis of diseases, surface properties of the red blood cells and methods, application in drug discovery and delivery, and imaging and sensing with the use of light scattering.

Section 3 presents a comprehensive overview of the structural and genetic mechanisms underlying the formation of biological photonic surfaces, pointing out the interdisciplinary nature of the research and its potential applications in both science and technology.

Section 4 discusses the interdisciplinary application of omics techniques to study complex biological photonic structures, which has implications for both basic biological research and the development of innovative materials and technologies.

Section 5 presents recent advances associated with bioinspired photonic materials for medical applications, considered as an answer to application problems of the currently used photonic materials including low biocompatibility and biodegradability, complex relationship between structure and optical properties (seen in dynamic adaptive color change), and limitation of scalability. Other presented challenges are closely related to the nature of materials and applications, e.g., in diagnostic and therapeutic methods.

Section 6 is dedicated to recent advances in the development and perspectives of optoelectronic sensors based on chiral nanomaterials including studies of the interplay between material chirality and biological responses, development of biosensing systems utilizing chiral nanomaterials, leveraging the sensitivity of nanooptics, and arising selectivity from the chirality of the materials. Moreover, examples of applications are shown.

Section 7 refers to the design and optimization of artificial bio-inspired photonic surfaces engineered to mimic the optical properties of natural materials, such as peacock feathers, butterfly wings, iridescent fruits, petals of plants, or the cuticle of some beetles. Challenges including the development of scalable fabrication techniques as well as stable and durable photonic structures precise control over the optical properties of photonic structures are discussed. Moreover, their potential applications in solar cells, optical sensors, and displays and cellulose liquid crystals are shown and as well as challenges referring to new areas of research and applications, e.g., development of photonic structures that can be tuned on demand to respond to changes in temperature or humidity.

Section 8 refers to the use of silica frustules as an example of a biomineralization process with possibilities for biomimetics to manufacture structures with photonic properties. Frustules can be manipulated or modeled to fully exploit their ability to handle optical radiation. This requires control of the individual components as well as all the interactions and conditions throughout the process. The first gene-edited cells with modified nanopore structures have been made based on genomics, and the potential to manipulate pore structure and properties has been proven by simple single-gene manipulations.

Section 9 considers bioinspired phantoms, which can be used in medicine to simulate selected properties of complex biomedical objects. Phantoms are designed and applied for mimicking biomedical objects, which can range anywhere from singular tissues, such as learning to perform skin stitches, or organs for performing procedures such as like gastro- or colonoscopy, up to whole systems such as the cardiovascular-respiratory system, or whole body parts—prosthetics, or organs, mainly for training purposes of medical procedures.

Section 10 refers to another group of bioinspired phantoms, which can be used in environmental protection to simulate selected optical properties of wastewater and enable calibration of fast analytical techniques—such as UV-Vis spectroscopy, fiber-optic interferometry, and Raman spectroscopy. Fast analysis of wastewater encounters significant challenges due to the highly complex biochemical composition, containing microorganisms, algae, fibers, salts, oils, and various contaminants. Developed phantoms accurately mimic the absorption, scattering, and chemical composition of real wastewater samples, thus facilitating reliable validation of sensors and analytical algorithms.

Section 11 highlights recent progress and the perspectives of Monte Carlo modeling of light interaction with biological tissues. This section outlines the forthcoming advancements in the polarized Monte Carlo approach, which will facilitate comprehensive and accurate modeling of polarized light interaction with biological tissues, considering the influence of bio-surfaces.

Section 12 concerns the possibility of the application of bio-inspired materials in optical fibers made for biomedical applications. Challenges and advances in science and technology are shown in cases of fibers made from cellulose and its modified forms as well as agar or agarose.

Section 13 discusses further development in the field of hyperspectral imaging for biological applications and clinical translation of hyperspectral imaging. In brief, hyperspectral imaging already showcases an excellent potential for becoming a valuable tool in a broad range of biological applications. Considerations regarding speed of data processing, acquisition, and novel numerical models of light-tissue interaction in the service of broader applicability are presented, along with examples of successful applications of the principles discussed.

Section 14 presents recent advances and the possibility of development in terahertz sensing and imaging of biological surfaces. Requirements on THz sources—quantum cascade lasers—are such as output power or frequency range are reviewed. Ways of improving the sensitivity of biosensors to trace amounts of samples by use of metamaterials (MMs), dedicated waveguides, and THz surface plasmon polaritons are presented.

Central unifying themes of this roadmap include surfaces, scattering phenomena, bioinspired materials, and their translation into sensing and imaging technologies. Requirements on bioinspired materials and measurement systems using them are reviewed. Considerations around the need to simplify the complexity and shrink the size of sensing systems are also discussed, important for the translation of devices into biomedical applications.

Overall, this roadmap aims to give a meaningful snapshot of developing topics in biophotonics. The field is much broader, hence this selection is not intended to be exhaustive, but aims to create a constructive picture of some of the major advances for the scientific community.

**Acknowledgments**

This Roadmap embraces the research vision and ambition of the European Cooperation in Science and Technology (COST) Action No. CA21159, understanding interaction of light—biological surfaces: possibility for new electronic materials and devices (PhoBioS).

## 2 Light Scattering by Biological Surfaces

**Section Authors**: Igor Meglinski (Aston University), Alexander Bykov (University of Oulu), and Maria Gritsevich (Finnish Geospatial Research Institute FGI and University of Helsinki)

The studies associated with light scattering by biological surfaces have a number of perspectives due to important applications in various fields such as biology, biophysics, biochemistry, and biomedical engineering. Some of the key perspectives include:

### 2.1 Characterization of Biological Structures

Light scattering can provide information about the morphology, composition, and organization of biological structures such as cells, tissues, and extracellular matrices. By characterizing the light prior to interactions and measuring the intensity, polarization, and spectral properties of the scattered light, researchers can extract information about the size, shape, refractive index, and optical properties of the structures. As has been recently demonstrated, an efficient non-contact, non-destructive technique can be developed when coupling light scattering measurements with acoustic levitation, allowing swift manipulation and characterization of the sample.^1^^,^^2^

### 2.2 Light Scattering for Disease Diagnosis and Health Monitoring

Light scattering can be used as a noninvasive and label-free tool for diagnosing various diseases, dysfunctions, and disorders. For example, changes in the light scattering properties of cells and tissues can be indicative of pathological conditions such as cancer, inflammation, and fibrosis. On a larger scale, light scattering is used in remote sensing techniques including sensor-based methods for the detection and identification of plant species and their diseases.^3^

### 2.3 Scattering by Complex Biological Surfaces

Light scattering is not limited to red blood cells (RBC) but is a fundamental phenomenon across a wide range of biological surfaces. Different classes of surfaces exhibit characteristic scattering behaviors that are highly relevant for diagnostics, sensing, and therapeutic monitoring.

#### 2.3.1 Epithelial and tissue layers

The layered architecture of skin, mucosa, and corneal tissues produces strong scattering due to refractive index mismatches and microscopic roughness. Polarization-sensitive measurements have shown potential for detecting early pathological changes such as precancerous lesions and fibrosis.

#### 2.3.2 Extracellular matrices and fibrous tissues

Structures such as collagen and elastin fibers create highly anisotropic scattering, which can be exploited to study tissue organization, remodeling, and disease progression. The orientation and density of fibrous proteins strongly influence both intensity and polarization properties of scattered light.

#### 2.3.3 Plant and microbial surfaces

Vegetation and microbial colonies present highly structured, often periodic or fractal surfaces that lead to distinctive scattering signatures. Applications include remote sensing of plant health and early identification of infections in crops and microbial biofilms.

#### 2.3.4 Interfaces and rough surfaces

Biological membranes and surface layers often exhibit nanoscale roughness and inhomogeneity, resulting in complex scattering patterns. These properties are increasingly explored using advanced techniques such as polarized light scattering, photonic nanojets, and super-resolution scattering–based imaging.

Together, these examples highlight that light scattering provides a versatile tool for probing the structure and function of diverse biological surfaces. RBCs serve as a particularly well-studied and accessible model, but the broader applicability of scattering methods underscores their relevance across multiple biological systems.

### 2.4 Red Blood Cells as a Model System for Probing Surface Properties

The investigation of the RBC surface utilizing light scattering has several perspectives, as it can provide important information about the physical and structural properties of RBCs. The key perspectives include the following:

#### 2.4.1 Biomedical research

Light scattering techniques such as dynamic light scattering (DLS) and diffusing wave spectroscopy (DWS) can be used to investigate the size, shape, and surface charge of RBCs.^4^ By analyzing the light scattering patterns of RBCs, researchers can obtain information about their structural properties and gain insights into the mechanisms of various biological processes such as blood coagulation and immune response.

#### 2.4.2 Blood disorders

Light scattering techniques are used to investigate the surface properties of RBCs in various blood disorders such as sickle cell disease, thalassemia, and malaria. By analyzing the light scattering patterns of RBCs, researchers can identify changes in their physical and structural properties, which can help in the diagnosis and monitoring of these disorders.

#### 2.4.3 Drug delivery

There are advantages to using light scattering techniques to investigate the interactions between RBCs and drug delivery systems. By analyzing the light scattering patterns of RBCs loaded with drug delivery systems, researchers can study the stability, release kinetics, and targeting efficiency of the drug delivery systems.

#### 2.4.4 Biophysics

Light scattering techniques can provide insights into the mechanical and physical properties of RBC membranes.^5^^,^^6^ By analyzing the light scattering patterns of RBCs under different mechanical stresses,^7^^–^^10^ researchers can study the deformation and mechanical properties of RBC membranes.

#### 2.4.5 Nanotechnology

Light scattering techniques can be used to investigate the interactions between RBCs and nanoparticles,^11^^,^^12^ which are being developed for various biomedical applications such as drug delivery and imaging. By analyzing the light scattering patterns of RBCs interacting with nanoparticles, researchers can study the mechanisms of nanoparticle uptake and toxicity.

Overall, the investigation of the RBC surface utilizing light scattering has broad perspectives. It provides important information about the physical and structural properties of RBCs, which can have implications for biomedical and biophysical applications as well as for newly emerging multidisciplinary studies where in-depth knowledge and characterization of RBCs are significant.

### 2.5 Light Scattering for Drug Discovery and Red-Blood-Cell-Based Delivery Systems

Light scattering can be used to study the interactions between drugs and biological surfaces, such as proteins, membranes, and cells. By measuring the changes in light scattering properties, researchers can determine the binding affinity, specificity, and mechanism of action of drugs, which can aid in drug discovery and development. There is also already a known novel medical paradigm in the framework of which RBCs have a great potential to be used as drug delivery carriers.^13^^,^^14^ In this paradigm, RBCs are loaded with therapeutic agents such as drugs, proteins, and nucleic acids, and are used as carriers to deliver these agents to target sites in the body. The advantages of using RBCs as drug delivery carriers are multifold. First, RBCs are biocompatible and biodegradable and can circulate in the bloodstream for several weeks without eliciting an immune response. Second, RBCs are highly deformable and can squeeze through narrow capillaries and reach target sites in the body that are inaccessible to other drug delivery systems. Third, RBCs can protect the loaded drugs from degradation and clearance by the body’s immune system, which can increase the drug’s circulation time and improve its therapeutic efficacy. RBC-based drug delivery has shown promise in preclinical studies for a variety of applications such as cancer therapy, gene therapy, and vaccine delivery. However, there are still challenges that need to be addressed before this technology can be translated into clinical use, such as improving drug loading efficiency, minimizing RBC clearance by the immune system, and ensuring the safety and efficacy of the loaded drugs. In recent studies, the mutual interaction of RBCs with drug-based nanoparticles (NPs) is investigated. With the combined use of optical tweezers and conventional microscopy modalities it has been explored the NP-induced alteration of hemorheological properties of RBC has been explored toward their use as drug carriers.^12^^,^^13^ Pronounced plasmonic response to IR laser light used for optical trapping was observed for some tested NP leading to RBC destruction via hyperthermia.^11^ In fact, the hemolytic activity of the NP and the effects arising at non-hemolytic levels such as RBC aggregation, membrane deformability, and morphology are required to be further investigated.

### 2.6 Future Opportunities in Scattering-based Imaging and Sensing

Light scattering can be used to develop imaging and sensing techniques for biomedical applications. This is especially powerful when combined with microscopy, laser scanning, or X-ray tomography to maximize understanding of the physical properties of the sample. For example, optical coherence tomography (OCT) and confocal microscopy use light scattering to produce high-resolution images of biological structures. Surface plasmon resonance (SPR) and localized surface plasmon resonance (LSPR) use light scattering to detect biomolecules and nanoparticles in real-time. Furthermore, although resolution in optical imaging poses a challenge to studying size scales below the diffraction limit, scattering-based imaging creates a pathway towards modern super-resolution techniques.^15^^–^^20^

### 2.7 Challenges and Future Directions

Despite substantial progress in understanding light scattering by biological surfaces, several fundamental and applied challenges remain to be addressed to advance the field:

#### 2.7.1 Multiscale complexity of biological systems

Biological surfaces span multiple scales, from nano-structured membranes to whole tissues, making accurate modelling and interpretation of scattering signals difficult.^21^ Developing integrative models that can link nanoscale molecular features with macroscale tissue architecture is a key challenge.

#### 2.7.2 Standardization and reproducibility

Current light scattering studies often rely on system-specific or laboratory-specific methods, hindering direct comparison of results. Establishing standardized experimental protocols and reference phantoms for biological surfaces would significantly improve reproducibility and enable cross-laboratory benchmarking.^22^^,^^23^

#### 2.7.3 In vivo translation

Much of the progress in scattering characterization has been made under controlled *in vitro* or *ex vivo* conditions. Translating these techniques to *in vivo* environments, where blood flow, tissue heterogeneity, and motion artifacts complicate measurements, remains a major hurdle.^24^^,^^25^

#### 2.7.4 Polarization and structured light

Although conventional scattering studies rely on intensity or spectral measurements, the use of advanced modalities such as polarized light, vortex beams, and orbital angular momentum (OAM) offers new opportunities to probe chirality, anisotropy, and subtle disease-related changes.^26^^,^^27^ Their robustness in turbid media and potential for label-free diagnostics require further systematic exploration.

#### 2.7.5 Computational and AI-driven analysis

The combination of physics-based forward models with machine learning approaches is increasingly seen as a pathway to solve inverse scattering problems in real time.^28^ This integration could enable accurate retrieval of morphological and biochemical parameters directly from complex scattering patterns.

#### 2.7.6 Clinical and technological translation

For scattering-based diagnostics and therapeutic monitoring to enter routine healthcare, devices must be cost-effective, portable, and user-friendly.^25^ Challenges include miniaturization of systems, robustness to patient variability, and ensuring regulatory approval for clinical use.^29^

Looking forward, progress will require closer integration between experimental optics, computational modeling, and clinical validation. Interdisciplinary collaboration, spanning photonics, biology, material science, and medicine, will be essential for transforming light scattering from a primarily research-focused tool into a widely adopted modality for biomedical diagnostics, monitoring, and therapy guidance.

## 3 Analysis of the structural/morphological/genetic mechanisms of formation of biological photonic surfaces

**Section Authors**: Mikhail Kryuchkov (University of Geneva), and Vladimir L. Katanaev (University of Geneva)

### 3.1 Status

Biological photonic surfaces reflect light in ways that can produce color and iridescence, or, in opposite, minimize reflection to achieve near transparency. These diverse light manipulations are achieved through interactions of light with nano- or microscopic structures covering the photonic surfaces. The structures can be arranged in various 2D and 3D patterns, including stripes, spots, and spirals, and can produce colors ranging from metallic blue and green to bright red and orange, or change light polarization.^30^^,^^31^

Structuring of the biological photonic surfaces is incredibly complex and varies among different organisms. However, some common features can be observed. One of the most important features is the size and shape of the nano/microscopic structures that create the color. These structures can be nanometers in size and are often arranged in patterns or layers to create an ordered structure.

Another essential aspect is the material that makes up the structures. In some cases, the structures are made up of proteins^32^ or chitin,^33^ whereas in others, they may be made of minerals such as calcium carbonate.^34^ The material used to create the structures can significantly influence the properties of the photonic surface.

The formation of biological photonic surfaces is a complex process influenced by various factors, including genetics. The genes that control the appearance of the structures that create the photonic surfaces are known as structural color genes. These genes code for proteins involved in producing and assembling the nano/microscopic structures that make a color. It was shown that a variety of factors, including environmental conditions and hormonal signals, can influence the expression of these genes. For example, the genes that control the formation of colorful feathers in male birds are activated by testosterone, resulting in the species’ bright colors.^35^

Organisms as small as bacteria can create photonic surfaces. Iridescent 1 (IR1) colonies *of Flavobacterium* strains display vivid, bright structural coloration due to their assembly into highly ordered patterns. By the transposon insertion method, it was possible to localize DNA regions and genes affecting the iridescence. Modifications of genes responsible for gliding motility, cell shape genes, stress response, and tRNA modification contributed most to the optical properties.^36^ A similar random mutation method with the usage of the mutagen ethyl methanesulfonate allowed researchers to find genes playing a role in forming the nanoridges in a plant cuticle. Diffraction gratings create the structural coloration of most angiosperm petals and depend on the cell wall and cuticle elongation.^37^ Mutagenesis and transgenesis showed that the cutin synthase 2 and wax biosynthesis transcription factors are necessary for cuticle folding and nanoridge formation.^38^

Not only the cuticle but the plant cell wall itself can create an iridescence of light. In the *Pollia* and *Margaritaria* genera, highly ordered cellulose microfibrils are used to generate multilayer reflectors. It was shown that such layers are formed in parallel to microtubules in the cortical arrays.^39^ Therefore, genetic changes affecting the cortical arrays or cellulose synthesis can be considered as potential ways to manipulate the plant multilayer reflectors. 

Another way plants form photonic structures is by arranging grana in specialized plastids called iridoplasts. Genes responsible for the thylakoid membrane formation and maintenance may be involved in the control over the arrangement of thylakoids and their grana.^37^ Insects produce a wide variety of photonic structures, from the colorful scales on butterflies’ wings^33^ and antireflective nanocoatings on their eyes,^40^ to the nanoridges on fireflies’ lanterns, or beetles’ helicoidal multilayered elytra that reflect linearly and circularly polarized light.^41^^,^^42^ In attempts to uncover the genetic mechanisms governing the formation of these patterns, mostly the chitin synthase and the regulatory genes initially emerged as promising candidates.^43^^,^^44^ These early attempts to find the genes responsible for the formation of nanostructures were based on the so-called growth model. According to this model, the nano/microstructures are formed with the help of the cell outgrowths—the microvilli. 

**Fig. 1:**
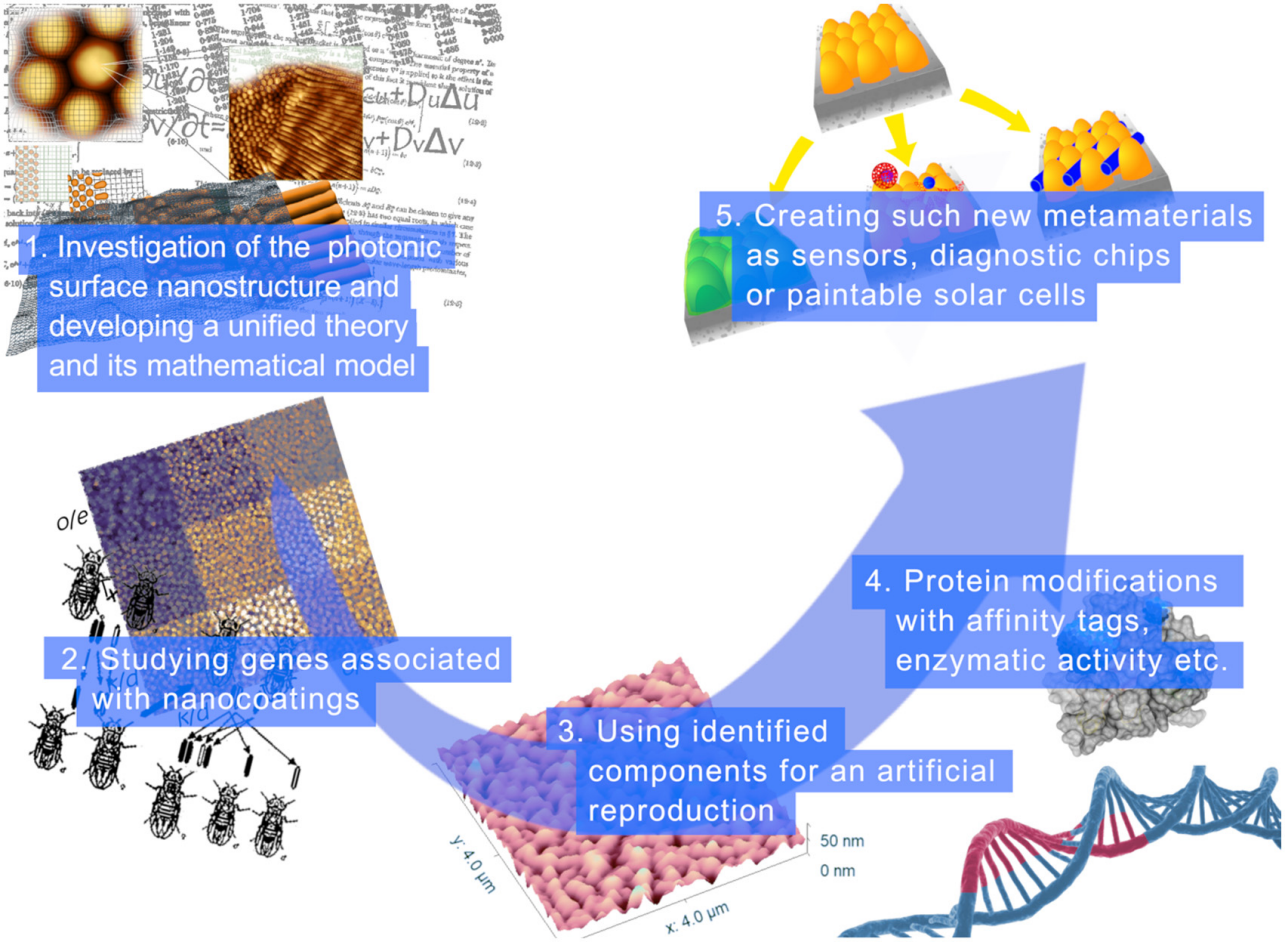
Milestones, both accomplished and still ongoing, in the multidisciplinary research based on insect corneal nanocoatings. Although these nanocoatings represent just one example of biological photonic surfaces, they have become a particularly tractable model system to integrate microscopy, genetics, optics, mathematical modeling, and materials science in a unified framework. This integrated approach serves as a roadmap for investigating the development and function of photonic structures in other organisms.

A paradigm shift was achieved with the realization that such structures can be formed by the self-assembly mechanism.^30^^,^^45^ The newly proposed reaction-diffusion self-assembly mechanism allowed researchers to predict the nature of these structures’ building blocks. Analysis of mutants, transgenesis, and RNA interference methods proved that the antireflective corneal nanoprotrusions are formed by the interaction between structural proteins such as retinins and wax-like molecules.^46^^–^^49^ As summarized in Fig. 1, insect corneal nanocoatings have become a model system to integrate microscopy, genetic tools, optical measurements, and mathematical modeling in a single framework, offering a roadmap that could also guide the study of other biological photonic surfaces. Following these advances, many examples of structural coloration whose development remains unknown, such as the changeable iridescence of cephalopods^48^ and the colorful plumage of birds,^32^ may hopefully be similarly decoded.

### 3.2 Current and Future Challenges

One of the main challenges in the genetic analysis of biological photonic surfaces is the insufficiency of tools and methods for these structures. Traditional genetic techniques, such as PCR and sequencing, are often limited in capturing their complexity. For example, it may be difficult to isolate the specific cells or tissues responsible for producing the photonic surface, or to extract genetic material from these small and spatially restricted sources without damaging the surrounding tissue, thereby risking contamination from adjacent, genetically distinct cell types. In addition, many of the genes regulating the surfaces may be expressed only during restricted developmental stages or in response to specific environmental cues, making it difficult to study them in a controlled experimental setting.

Another challenge in the genetic analysis of biological photonic surfaces is the need for interdisciplinary collaboration. Indeed, the understanding of the genetic mechanisms behind these structures requires expertise in genetics, cell biology, optics, materials science, and mathematics. However, these fields are typically isolated, and researchers may not have the necessary background or training to collaborate effectively. Figure 1 illustrates how combining these disciplines in the context of insect corneal nanocoatings has already yielded significant insights and offers an example of how such integrated approaches can accelerate both fundamental discoveries and technological applications.

### 3.3 Advances in Science and Technology to Meet Challenges

Despite the challenges, recent advances in technology and in interdisciplinary collaboration have made it possible to gain new insights into the genetic mechanisms behind biological photonic surfaces. For example, new imaging techniques such as localization atomic force microscopy, super-resolution or expansion microscopy^49^^–^^51^ allow researchers to visualize these surfaces’ structure, composition, and development. This can help in identifying the specific cells/tissues responsible for the structures’ production and to track changes in them over time. In addition, advances in genome sequencing^52^ and single-cell transcriptomics^53^ have enabled identifying the specific genes and proteins involved in producing these structures. By manipulating these genes, researchers can test their function in the production of the surfaces and identify the specific pathways involved. New gene editing and manipulation tools have made it possible to test the function of specific genes and proteins in producing these surfaces. For example, CRISPR-Cas9 technology allows editing the cells’ DNA sequence to create particular mutations or knockouts.^54^ This can help identify the specific genes or mutations responsible for the surface’s production. In addition, advances in materials science have made it possible to create synthetic materials that mimic the properties of biological photonic surfaces. By studying the genetic mechanisms behind these surfaces, researchers can develop new materials and technologies that have a wide range of applications, from fashion to security.^55^^,^^56^

### 3.4 Concluding Remarks

Genetic analysis of biological photonic surfaces presents a unique set of challenges and a wealth of opportunities for understanding and utilizing these fascinating structures. By taking a multidisciplinary approach that combines expertise in genetics, cell biology, optics, and materials science, researchers can overcome these challenges and unlock the potential of these structures for a wide range of applications. Studying the genetics of photonic surfaces is a challenging task, but a variety of techniques have been developed that can help in this endeavor. The study of the genetics of photonic surfaces may lead to many potential applications. One of the most promising is the development of new materials that can mimic or even reproduce^57^ the properties of biological photonic surfaces. These materials could be used in a variety of applications, including the development of new optical devices and sensors.^42^ Another area of research is the study of the environmental factors that influence the expression of the structural color genes. This could have important implications for understanding how organisms adapt to changing environmental conditions. The study of the genetics of photonic surfaces is still in its early stages, but there are many exciting prospects for future research. One area of research that is likely to be important is the study of the evolution of photonic structures. By comparing the structural color genes of different species, researchers can gain insights into how these structures have evolved over time.

**Acknowledgments**

This work was supported by the Swiss National Science Foundation (Grant No. 310030_192527) to V.L.K.

## 4 Omics Analytical Techniques to Reveal the Composition of the Surface Materials Building Up the Photonic Structures

**Section Authors**: Mikhail Kryuchkov (University of Geneva), and Vladimir L. Katanaev (University of Geneva)

### 4.1 Status

Biological photonic surfaces are biological structures that manipulate light utilizing specialized tissues, cells, composite materials, or molecules. These complex surfaces can be found in various organisms, from bacteria to vertebrates. The light transformation produced by these surfaces can range from iridescence to anti-reflectance and is often used for multiple purposes, such as camouflage,^58^ mate attraction, navigation,^59^ and signaling. For example, the wings of butterflies and moths are covered by tiny scales that produce colors through the interaction of light with specialized structures called photonic crystals. These crystals comprise layers of proteins^60^ and other molecules that reflect and refract light, creating the vibrant colors and patterns we admire.^61^ Similarly, the skin of some fish, such as the mandarin fish, contains specialized cells called chromatophores that can change color in response to different stimuli, such as light or temperature.^62^ Understanding the mechanisms behind biological photonic surfaces is essential for various reasons. For one, these surfaces can provide insights into the evolution and diversity of life on Earth. By studying the different types of photonic structures found in different organisms, we can better understand how they evolved and adapted to different environments. In addition, understanding biological photonic surfaces can have practical applications, e.g., in developing new materials and technologies. For example, the photonic crystals found in butterfly wings have inspired the development of new types of optical sensors and displays.^63^ Similarly, the chromatophores found in some cephalopods have inspired the development of new kinds of camouflage materials.^64^ Omics analytical techniques are tools and methods used to identify and study the different components of biological systems, including genes, proteins, metabolites, and more. There are several omics techniques, each focusing on a different aspect of biological systems. Here, we try to characterise the state of the art of all omics approaches individually.

#### 4.1.1 Proteomics

Proteomics studies an organism’s or tissues’ entire complement of proteins, including their structure, function, and interactions. By analyzing the proteins present on a biological photonic surface, insights can be gained into the molecular mechanisms behind its color and pattern production. Recent developments in proteomics analytical techniques have made it possible to reveal the composition of the surface materials building up the photonic structures. Mass spectrometry is one of the most commonly used proteomics analytical techniques for identifying the composition of photonic surfaces. Mass spectrometry is a powerful tool for studying proteins and peptides in complex mixtures, as it can identify and quantify the individual components within a sample. By analyzing the mass-to-charge ratio of proteins and peptides, mass spectrometry can provide information on the amino acid sequence, post-translational modifications, and overall composition of these molecules. This information can pinpoint the specific proteins and peptides on the surface of photonic structures. Exactly this method was used for the identification of a brochosome’s composition. Brocosomes are nanostructured granules secreted by leafhoppers. They provide many functions, including the ultra-high antireflectivity needed for camouflage.^65^ Mass spectrometry analysis of these microparticles showed that they consist of a particular type of structural protein named brochosomins.^66^ The protein nature of insects’ corneal antireflective nanostructures was discovered similarly.^67^^,^^68^ Another example of the purely proteomic identification of the reflective tissue’s composition is the reflectin proteins.^69^ These proteins are deposited in the flat, highly ordered structures in cephalopod iridophores, playing a role in camouflage and communication. In conclusion, proteomics gives us valuable tools for revealing the composition of the surface materials building up the photonic structures; however, the proteomic analysis’s most direct limitation is the need for the protein database. Such a dataset could be made from the DNA sequence, which requires the genomic investigation of species.

#### 4.1.2 Genomics

Genomics is the study of an organism’s entire genetic makeup, including all of its genes and other DNA sequences. By analyzing an organism’s genome, it is possible to gain insight into its evolutionary history, as well as its physical and behavioral traits. In the context of biological photonic surfaces, genomics can be used to identify the genes responsible for producing the specialized structures and molecules that create the colors and patterns on these surfaces. The DNA analysis of the organisms that produce these surfaces can give insights into the chemical and structural properties of the materials themselves. For example, genomics has been used to study the bioluminescent organs of certain cephalopods composed of complex arrays of photonic crystals. By sequencing the genomes of these organisms, the genes responsible for producing these crystals and the proteins involved in their formation were identified.^70^ Overall, genomics analytical techniques have opened up exciting new avenues for studying the composition and properties of photonic surfaces. By combining these techniques with other tools such as microscopy, spectroscopy, and computational modeling, a deeper understanding of how these materials work and how they can be used in a variety of applications can be gained.

#### 4.1.3 Transcriptomics

Transcriptomics refers to the study of the transcriptome, the complete set of RNA transcripts produced by an organism’s cells. It involves the analysis of RNA molecules to provide information about gene expression, alternative splicing, and other cellular processes. Transcriptomics has emerged as a powerful tool for understanding the composition of materials, including those used to build photonic structures. A better understanding of their composition can be obtained by identifying the RNA transcripts of the cells that make up photonic surfaces. For example, identification of the genes that are responsible for producing the proteins that make up these surfaces and also a determination of how these genes are regulated can provide insights into the cellular processes that are involved in building these structures.^71^ One of the key advantages of using transcriptomics to study photonic surfaces is that it can provide a more comprehensive view of these materials than other analytical techniques. Traditional methods, such as electron or atomic-force microscopies, can provide detailed information about the physical structure of a material. However, they may not reveal the full complexity of the material, including the specific proteins and other molecules present. In addition, transcriptomics can be used to study photonic surfaces under a wide range of conditions. The transcriptome of cells that are grown under different temperatures, pH values, or other environmental factors can be analyzed. For example, the artificial propagation conditions were investigated as a crucial factor for the grouper fish coloration.^72^ This can provide insights into how these materials behave under different conditions, which can be important for optimizing their performance in various applications. Overall, transcriptomics is a powerful tool for studying the composition of photonic surfaces. By providing insights into the genes and cellular processes involved in building these materials, transcriptomics can help optimize their performance for a wide range of applications.

#### 4.1.4 Metabolomics

Metabolomics is a rapidly growing field that involves identifying and quantifying small molecules within biological systems. Metabolomics techniques have expanded beyond traditional biology and have been used to study a wide range of materials, including photonic surfaces. These techniques involve using mass spectrometry and nuclear magnetic resonance spectroscopy, among others, to identify the small molecules present in the surface materials. This information can then be used to better understand the mechanisms behind the formation of the photonic structures and potentially even replicate them for applications in various industries. One of the key advantages of using metabolomics techniques to study photonic surfaces is the ability to understand the system comprehensively. Metabolomics techniques can identify a wide range of small molecules that may be present in the surface materials, including lipids, amino acids, and sugars. Overall, the use of metabolomics analytical techniques in the study of photonic surfaces has provided valuable insights into the composition and formation of these structures. Understanding differences between metabolomics pathways can help explain the appearance of bright colors.^73^ As the field of metabolomics continues to expand and evolve, it is likely that these techniques will be further refined and improved, leading to even greater insights into the complex biological and non-biological systems that make up our world.

#### 4.1.5 Metagenomics

Metagenomics studies the microbial communities in a particular environment, including those on biological surfaces. By analyzing the microbial communities on a natural photonic surface, we can understand how they interact with each other and the host organism and their role in color and pattern production. For example, algae stimulation of structural coloration was found for the iridescent bacterial colonies.^74^

#### 4.1.6 Phenomics

Phenomics is an emerging field of science that uses various analytical techniques to analyze the composition of biological materials and their interactions with the environment. One of the critical applications of phenomics is in the study of photonic surfaces. Phenomics analytical techniques are essential in revealing the composition of the surface materials building up the photonic structures. These techniques include but are not limited to electron microscopy, atomic force microscopy, Fourier transform infrared spectroscopy, and photometry. These techniques help to provide detailed information on the surface morphology, chemical composition, and crystal structure of the photonic surfaces and their dependence on different factors. Such an approach was used for the butterflies’ phenotypic plasticity investigation and the origin of the color differences. It included several spectroscopy measurement methods compared with electron microscopy techniques.^75^

### 4.2 Current and Future Challenges

One of the challenges in omics analysis for photonic structures is the limited availability of properly prepared samples for experimental analysis. Photonic structures are complex and require extensive isolation from the non-photonic tissues for proper investigation. There is a need to develop new experimental techniques to accurately measure the molecular composition of photonic structures from limited isolates. Another challenge is the integration of different omics analytical techniques. Photonic structures comprise various molecules such as proteins, lipids, and nucleic acids. Each of these molecules has its unique omics analysis technique, and integrating these techniques can be challenging. Moreover, analyzing photonic structures requires identifying trace amounts of molecules, which can be challenging to detect using traditional omics analytical techniques. Therefore, there is a need to develop new omics analytical techniques that can detect trace amounts of molecules accurately. Finally, the analysis of photonic structures requires the identification of the spatial distribution of molecules. The spatial distribution of molecules can impact the properties of photonic structures. However, traditional omics analytical techniques do not provide this information. Therefore, there is a need to develop new omics analytical techniques that can provide information on the spatial distribution of molecules.

### 4.3 Advances in Science and Technology to Meet Challenges

The future of omics analysis for photonic structures lies in developing new experimental techniques and integrating different omics analytical techniques. One of the advancements in omics analysis is the use of nanotechnology. Nanotechnology can be used to create sensors that can accurately detect trace amounts of molecules.^59^^,^^63^^,^^76^^,^^77^ Moreover, nanotechnology can be used to develop new experimental techniques that can accurately measure molecules’ spatial distribution. Another advancement in omics analysis is its integration with machine learning. Machine learning can be used to develop accurate computational models that can accurately predict photonic structures’ properties.^78^ Moreover, machine learning can be used to accurately identify molecules’ spatial distribution. The future of omics analysis for photonic structures also lies in collaborative research efforts. It can bring together experts in different fields to develop new omics analytical techniques and experimental techniques. Moreover, collaborative research efforts can lead to the development of new applications for photonic structures.

### 4.4 Concluding Remarks

Omics analysis is essential in understanding the molecular composition of photonic structures. However, there are still many challenges in omics analysis for photonic structures, such as the limited availability of experimental data and the integration of different omics analytical techniques. The future of omics analysis for photonic structures lies in developing new experimental techniques, integrating omics analysis and machine learning, and collaborative research efforts. Therefore, there is a need for more research in these areas to unlock the full potential of photonic structures.

**Acknowledgments**

This work was supported by the Swiss National Science Foundation (Grant No. 310030_192527) to V.L.K.

## 5 Bioinspired Photonic Materials for Medical Applications

**Section Authors**: Brindusa Dragoi (Regional Institute of Oncology, TRANSCEND Research Center and Al. I. Cuza University) and Nicolina Pop (Politehnica University of Timisoara)

### 5.1 Status

Biophotonics plays a significant role in advancing disease diagnostics and therapy, particularly using noninvasive or minimally invasive optical techniques.^79^^,^^80^ Because biophotonics has found applications for diagnostic and treatment, it can be divided into diagnostic biophotonics and therapeutic biophotonics. The primary aim of diagnostic biophotonics is to detect disease as early as possible, ideally before any symptoms occur. Although many biophotonic sensors operate in a single dimension, certain photonic techniques, such as optical coherence tomography (OCT), allow for the assessment of multidimensional data.^81^ For this particular case of OCT, it is preferred over the non-photonic approaches, like impedance spectroscopy also operates across multiple dimensions, due to certain advantages. Therefore, OCT generates high-resolution 3D images and allows the visualization of the tissue structures in real time.^82^ On the contrary, the non-photonic impedance spectroscopy generates useful data on the electric properties of the tissues and cells for diagnostic purposes.^83^ To go deeper into the characteristics of biological tissues, Bagnaninchi et al. combined Fourier domain optical coherence tomography (FDOCT) and impedance spectroscopy (IS) into a single instrument to investigate the parameters involved in tissue formation, simultaneously obtaining structural and electrical information.^84^ In addition, photonics can be employed for the effective and long-term monitoring of certain diseases, such as cancer, which is the most investigated disease in nanophotonics.^85^^,^^86^ Therapeutic biophotonics uses optical radiation for treating diseases by altering the chemical and/or mechanical properties of biological matter, which leads to cell death, as is the case with cancer. In addition, the functions of the cells can be altered photochemically, photothermally using optical radiation at a certain frequency.

Due to its advantages, nanotechnology has been integrated into biophotonics, aiming at identifying relevant biomarkers for diseases. Hence, a new field, that is, nanobiophotonics, was produced, which deals with light-matter interactions on the nanoscale, offering new opportunities for personalized diagnosis and treatment to each personal molecular profile.^87^^,^^88^ Nanomaterials used in biophotonics, usually metals or semiconductors, with a high surface-to-volume ratio, exhibit unique optical, chemical, and electronic properties. These properties make them attractive candidates as biosensors and nanocarriers for the detection of diseases, real-time monitoring of molecules, and therapy.^89^ Photonic nanomaterials emit, detect, manipulate, or control light, offering information on the interaction between optical radiation and biological matter at the sub-wavelength scale.^85^ As a result, the introduction of nanomaterials into biophotonics changed the paradigm of biophotonics for diagnostic and therapy, enabling a more precise and flexible detection. They are the forefront of the development of several powerful technologies for molecular diagnostics, such as localized surface plasmon resonance, surface enhanced Raman spectroscopy, NIR-based fluorescence, optical coherence tomography, photoacoustic imaging, two-photon luminescence, plasmonic photothermal therapy, and photodynamic therapy for treatment as well as for combined diagnostics/therapeutics (theranostics).^79^ Although nanobiophotonics is a very recent multidisciplinary field of research for medical applications, it has advanced fast and shows potential for diagnosis and therapy of many diseases. The achievements made so far are explained by the fact that nanobiophotonics can access tissues and cells at previously unreachable levels. Yet, all studies are in preclinical or clinical stages, none of the tools of nanobiophotonics being used in the daily practice in hospitals due to the scientific, technical, and regulatory barriers that need to be addressed in the (near) future.^79^^,^^90^^,^^91^

### 5.2 Current and Future Challenges

During the last years, the design and synthesis of biophotonic materials and their applications in the medical field have both evolved. They could be considered an answer to the low biocompatibility and biodegradability of the currently used photonic materials, such as silica or organic polymers.^92^ However, there are several challenges needing solutions because many things in the biophotonic systems are not yet understood. The good part is that what is understood has already impacted the potential medical applications at the fundamental level, encouraging further investigation into this route. The first challenge in this area would be related to the synthesis of materials with optical properties inspired by nature. Usually, the nanostructures existing in living organisms, which exhibit special optical properties, are gathered into complicated morphologies whose reproduction in the laboratory is not very straightforward. Chemists, materials scientists, and specialists in computing should put together their know-how to reproduce such structures by the currently available synthesis methods. An in-depth understanding of the complex relationship between structure and optical properties is also needed to design and synthesize novel composite biophotonic nanostructures with new and reproducible optical effects, and thus innovative applications, which implies a closer collaboration with biologists and physicists. In this context, dynamic, adaptive color change, as is the case with cephalopods, is by far related to one of the most complex structures to be designed by man. The next step in the development of these materials is scalability or translating the results of the research in nanobiophotonics into next-generation devices for clinics, which involves the identification of an appropriate synthesis method in terms of yield, reproducibility, and cost-efficiency, as well as regulatory challenges.^93^ Alternative solutions could be the employment of smart biocompatible materials either found in nature, such as chitin,^94^ green fluorescent protein,^95^ or synthesized in the laboratory at a high purity, such as two-dimensional layered double hydroxides, with tunable structural properties and optical effects depending on the application, and with larger chances to be translated into the clinic.^96^^,^^97^ Other challenges are closely related to the nature of materials, the used technique, and the application. For instance, the performance of the spectroscopic techniques coupled with a nanophotonic material strongly depends on the size, shape, dispersion, and stability against aggregation.^98^^,^^99^ Plasmonic photothermal therapy (PPTT) uses gold-based nanoparticles to induce the death of the cancer cells, and it is considered a good alternative to traditional treatments, such as chemotherapy, radiotherapy, and surgery. Although gold nanoparticles employed in PPTT are not intrinsically bio-inspired, bio-inspired design approaches are used in their synthesis to improve their biocompatibility and stability in biological environments. For example, Zhou et al. synthesized BSA-bioinspired gold nanorods loaded with R837 immunoadjuvant for melanoma treatment by combining PTT and immunotherapy, with a high potential for clinical practice.^100^

Despite the benefits of PPTT, the method is not ready yet to be largely applied in daily medical practice due to several challenges that require appropriate investigations, such as long-terms effects of nanoparticles in the human body, standardization of the nanoparticles synthesis, and limited penetration of the light to <1 cm.^101^ Surface enhanced Raman spectroscopy (SERS) is another technique with real potential for daily use in clinics, but it currently faces several challenges that does not allow going beyond the stage of clinical trials.^102^ SERS depends on the metal surface but also on the proximity of these nanostructures, whereas the penetration length is <1 cm. Due to the obtained cross-sectional and high-resolution three-dimensional images of tissue microstructure, optical coherence tomography (OCT)—a light-based imaging technique in biophotonics—has already widespread applications for many medical areas.^82^ Still, OCT has a limited imaging depth of ∼2–3 mm, making it a proper tool for superficial tissues.^103^^,^^104^

To extend the imaging depth and thus the range of medical applications, the incorporation of nanoparticles into OCT is a smart approach due to the controllable size, shape, and composition, which impact their optical properties.^105^ Therefore, by combining the resolution of OCT with the ability of nanoparticles to act as contrast agents, the biological structures are better visualized. However, nano-sensitive OCT is at an early stage of development, but it is a promising area of research with a lot of potential for cellular-level diagnostics. So far, silver and gold nanoparticles are reported for OCT in dental medicine, visualization of blood vessels in tumors and healthy tissue, and lymph vessels, as well as tumor-associated structural changes.^106^^–^^108^

Bio-inspired methods for the synthesis of nanoparticles for OCT involve the use of natural templates or biological systems, making them inherently more biocompatible and less toxic whereas the method is considered simple, cost-effective, and environmentally friendly.^109^^,^^110^ Yet, the nanoparticles are synthesized in different labs using various sources and experimental conditions, resulting in a range of sizes and shapes, ultimately impacting the localized surface plasmon resonance (LSPR), the phenomenon that improves the contrast in nano-sensitive OCT. Another challenge that should be overcome is related to the surface functionalization of gold nanoparticles with biomolecules, a strategy used to increase the precision of diagnostics. Studies revealed that the organic part of the hybrid nanoparticles suffers thermal decomposition and photo-oxidation, altering the LSPR signal. Molecular fouling on the nanostructures and the inability of LSPR to distinguish analytes of similar functionality require additional fundamental research for optimization.^111^

Overall, it appears that beyond the inherent limitations and challenges of the techniques themselves, the real need is the development of more biocompatible nanomaterials under standardized experimental conditions to properly compare the results obtained in various research groups. It can be affirmed that all identified challenges are in fact stimulating opportunities and an open avenue for advancing fundamental and translational research in (bioinspired) photonic materials for medical applications.

### 5.3 Advances in Science and Technology to Meet Challenges

Observing the cellular processes, which have extremely high dynamics, at super-resolution is an enormous challenge in the biomedical field. Although the concept of high resolution and depth of focus in microscopy dates back to 1970’s,^112^ its development through various techniques has led into the development of super resolution fluorescence microscopy (SRM), for which the Nobel Prize was awarded in 2014. This is the critical time that significantly accelerated its application in cell biology, allowing the study of living cells at the molecular level and observing single proteins with unprecedented detail has become possible, revolutionizing diagnostic approaches and intensifying research on this topic. This technique pushed the limits of the detection till a spatial resolution of ∼20–100 nm. Such detailed and in-depth characterization allows better understanding of the mechanisms of diseases, guiding thus the therapy, a critical step forward in precision medicine and personalized medicine.^113^^–^^115^

The current SRMs are roughly separated into three categories, including stimulated emission depletion (STED), (saturated) structured illumination microscopy [(S)SIM], and single-molecule localization microscopy (SMLM) represented by photo-activated localization microscopy (PALM) and stochastically optical reconstruction microscopy (STORM), which provides the resolution needed to go deeper insight into the biochemical mechanisms behind the diseases.^116^ However, the results obtained so far are on animal models; adoption of such a technique in daily medical practice depends on many regulatory aspects. These come together with some challenges of the SRM itself, because, realistically, no technique can provide exhaustive and ideal images/results. Still, the *in vivo* results are superior to those obtained by other currently existing techniques, highly encouraging the adoption of super-resolution fluorescence microscopy techniques in clinics. One of the drawbacks of the SRM is the necessity of using fluorescent probes with higher quantum yields.^116^^,^^117^ Luminescent particles in nanoscale size with high photostability and increased photophysical properties, such as gold, carbon dots, quantum dots, polymer dots, silica, nanodiamonds, and upconversion nanoparticles, hold great promise for the SRMs to improve image quality and resolution.^116^^,^^118^^,^^119^ Yet, they need to simultaneously exhibit biocompatibility, the capability to escape the control-defense mechanisms of the body, and a specific target. Therefore, nanoparticles with a nature-inspired design having particular structures and optical effects or synthesized using biological methods are potentially more appropriate to meet these challenges. Although most of the particles used for imaging techniques such as SRM are synthesized by conventional chemical approaches, the interest in developing bio-inspired nanoparticles is already observed. For instance, Zhi et al. employed malic acid, a green and inexpensive organic compound, as the carbon source to synthesize highly fluorescent bio-inspired carbon dots *via* a rapid microwave-assisted heating treatment, which exhibited enhanced spatial resolution in super-resolution localization imaging experiments.^120^ The neem extract roots were an excellent source for obtaining biocompatible graphene quantum dots (GQDs) via a hydrothermal method. Apart from exhibiting excellent biocompatibility, photo-stability, up-conversion, and continuous full-color emission, CQDs allowed for the visualization of lysosomes through structured illumination microscopy, a type of super-resolution fluorescence microscopy.^121^ The field is in its early stages, but these bioinspired nanoparticles are critical for the future of SRM, and in general for the high-resolution techniques used in diagnostic and/or therapy. This achievement is also needed because the nanoparticles with optical properties used in the SRM-based techniques are primarily based on noble metals, which are very expensive, making the final cost of the procedure rather unaffordable on a larger scale. Expanding the range of nanoparticles to include other chemical compositions, as mentioned above, also entails other disadvantages, such as toxicity, the need for functionalization, as well as negative impact on the environment due to the use of toxic solvents, chemicals, or high temperatures. These, combined with the cost of production, lead to the need to develop bio-inspired nanoparticles as a promising alternative for fluorescent probes for SRM. To advance the clinical applications of the bioinspired nanoparticles and SRM, and enable more timely, accurate, and personalized diagnosis and more effective therapy, another concern should be considered. All clinical trials in this field of research are conducted under different protocols, even for the same technique, which makes it difficult to draw a clear conclusion regarding the benefits and shortcomings of a particular method of diagnostic and or therapy. Therefore, there is a real need for standardization of the protocols used in, usually, multicenter and large-scale clinical trials.

### 5.4 Concluding Remarks

Biophotonics is a very generous field of research and innovation for medical applications focused on diagnostics and therapy, which has evolved significantly in the last at least 10 years, particularly after including nanomaterials in the methodology and breaking the physical limits imposed by classical optical microscopy. As a result, taking advantages of the light—bio interactions and unique optical properties of nanomaterials, there is a lot of unprecedentedly potential concerning the improvement of the molecular diagnostic and therapeutic approaches, which allow timely and more accurate detection of the disorders at the molecular level, the first stage of the diseases, and a tailored therapy, as critical aspects of the precision medicine and personalized medicine. However, despite the high performance in terms of spatial and time resolution and efficacy obtained so far, there is still room for investigation and innovation. Many of these techniques, or bio-inspired nanomaterials with special optical effects, are still in preclinical investigations or clinical trials. In addition, standardization of both the synthesis protocols for bioinspired photonic materials and clinical trial protocols is needed to speed up the translation from the bench to the bedside. Therefore, common efforts of specialists in various scientific areas are critical to advance on this route. It is expected to develop more precise, reliable, and efficient biphotonic technologies and devices for diagnostic and therapy, especially for non-communicable and challenging diseases, such as cancer.

**Acknowledgments**

This work was supported by the following projects: WIDESPREAD-06-2020-ERA Chairs/H2020 ERA-Chair projects—(Grant No. 952390/2020), Ministry of Education and Research, CNCS/ CCCDI-UEFISCDI (Grant No. PN-III-P3-3.6-H2020-2020-0105/35/2021), and COST Action No. CA21159.

## 6 Chiral Nanomaterials-Based Optoelectronic Sensors

**Section Author**: Junyoung Kwon (Pukyong National University)

Chiral nanomaterial-based optoelectronic sensors have recently emerged as attractive, bio-inspired sensing platforms that exhibit unique interactions with biosystems.^122^ Chirality is one of the key features of biosystems, evident in the chiral nature of fundamental building blocks such as amino acids and sugars. Moreover, these chiral building blocks give rise to complex chiral structures on a larger scale, encompassing proteins, DNAs, and even human hands.^123^ It has been reported that the molecular handedness of chiral medicines can impact their therapeutic effects depending on the enantiomer type. For instance, thalidomide, a sedative used to treat insomnia in the late 1950s, exhibited sedative effects for the R-type thalidomide, whereas the S-type thalidomide caused severe teratogenic effects.^124^ These examples have inspired scientists to explore the interplay between material chirality and biological responses, leading to the development of biosensing systems utilizing chiral nanomaterials.^125^^–^^127^ By leveraging the sensitivity of nanooptics enabled by nanomaterials and the distinct selectivity arising from their chirality, chiral nanomaterial-based optoelectronic sensing systems offer promising possibilities for the detection and analysis of biomolecules.^128^^,^^129^

### 6.1 Recent and Future Challenges

Despite the promising possibilities for developing highly sensitive chiral biosensors, their practical utilization in sensing devices has been hindered by their low g-factor—a dimensionless dissymmetry factor defined as $2(A_{L}-A_{R})/(A_{L}+A_{R})$ where $A_{L}$ and $A_{R}$ are the absorbances for left- and right-circularly polarized light, respectively—resulting in negligible changes in circular dichroism signals.^130^ Furthermore, the length scale mismatch between chiral biomolecules and circularly polarized light, which has relatively large wavelengths compared with the dimensions of biomolecules, is also a factor contributing to decreased sensitivity of the chiral biosensor.^131^ To address these challenges, nanoscale engineering of chiral nanomaterials, as well as assembly techniques beyond the nanoscale, has recently been introduced.^130^^,^^132^

### 6.2 Advances in Science and Technology to Meet Challenges

**Fig. 2:**
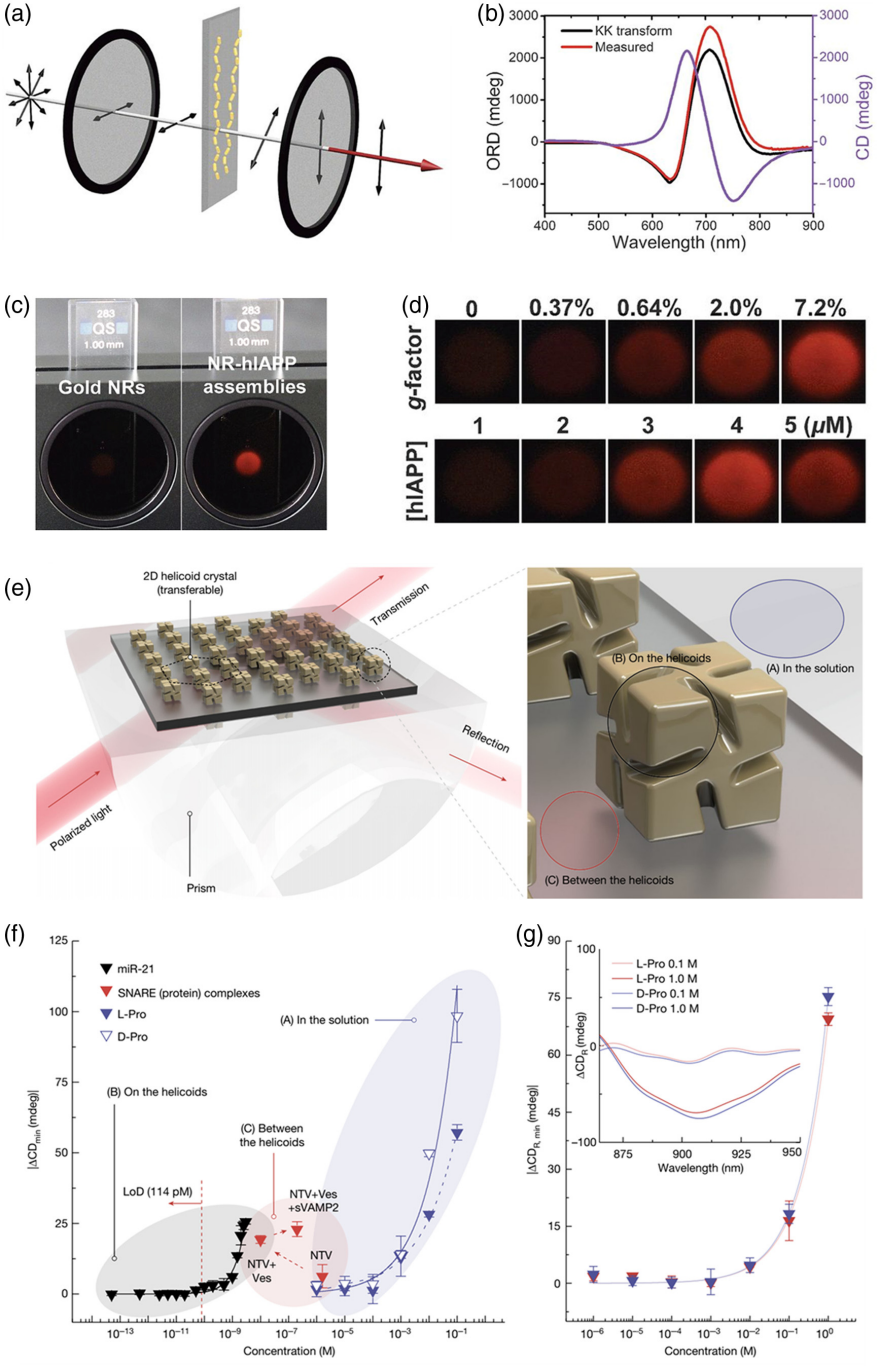
Chiral nanomaterials-based optoelectronic sensing systems. (a)–(d) Amyloid peptide sensing system based on the accelerated assembly of liquid crystal-like nanorod-hIAPP nanohelices. (a) Schematics for cross-polarization optical setup. (b) Optical rotatory dispersion (ORD) spectrum of nanorod-hIAPP assemblies from the measurement and Kramers-Kronig (KK) transformation to the corresponding CD spectra. (c) Digital photographs of pure NR and nanorod-hIAPP assemblies monitored, showing the red light transmitted under cross-polarized conditions. (d) Photographs of the nanorod-hIAPP assemblies with different g-factors and coassembled from variable hIAPP concentrations. Reproduced with permission from Ref. 130. (e)–(g) 2D helicoid crystal–based sensors. (e) Schematic illustration of a 2D helicoid crystal for both transmission and reflection modes, which is effective for various positions of biomolecules. (f) Absolute minimum value of the collective CD change induced by various biomolecules. (g) Change in reflection CD spectra (inset) and absolute value of ΔCDR at the minima acquired with a reflective SPR sensor. Reproduced with permission from Ref. 132.

In 2021, Lu et al. adopted long-range organization to enhance the chiroptical response of achiral Au nanorods. They achieved this by assembling the nanorods into cholesteric liquid crystal-like helices, resulting in a 4600-fold increase in the g-factor.^130^ The authors discovered that co-assembly of Au nanorods with the helical structure of the human islet amyloid polypeptide (hIAPP) allowed for precise control of geometry, including gap distance between Au nanorods, nanohelix pitch length, and number of turns [Figs. 2(a)–2(d)]. This precise control led to a high asymmetry factor of 0.1. Due to the high asymmetry factor and the time-dependent structural changes, the system exhibited a nontrivial optical rotatory dispersion signal that depended on the amount of amyloids present. This feature made the system suitable for screening inhibiting drugs, such as EGCG and D-NFGAIL, which bind to amyloids and inhibit their self-assembly.

The Nam research group reported the formation of 3D chiral Au nanostructures using amino acids and peptides through the enantioselective interaction of high-Miller-index surfaces in 2018.^133^ These structures exhibited a high g-factor of 0.2. Subsequently, the same research group utilized these chiral Au nanostructures to develop a 2D biosensor.^132^ The biosensor involved the deposition of a 2D array of helicoid Au nanoparticles, enabling the quantitative determination and in situ monitoring of molecular chirality during DNA-RNA hybridization and protein folding, even at very low concentrations down to $10^{-12}\;M$ (see Figs. 2(e)–2(g)). Notably, the controlled alignment of the helicoid Au nanoparticles into a periodic hexagonal pattern on a nanopatterned polymer surface, along with the collective resonances, facilitated the detection of low concentrations.

To sum up, chiral nanomaterial–based biosensors have emerged as promising techniques due to significant advancements in highly dissymmetric chiral nanomaterials. However, despite recent reports on chiral nanomaterial–based biosensors, these studies have primarily utilized pure analyte solutions. As a result, the potential interference from other chiral biomolecules when working with real biological samples, such as blood, saliva, and urine, remains unknown. To further advance this field, it is crucial to develop chiral nanomaterial–based biosensors that are tested with real biological samples while employing well-defined mechanisms. Besides, exploring the unique chiral interactions between chiral nanomaterials and chiral analytes can unveil opportunities to develop highly sensitive biosensors that surpass the detection limits achieved with achiral nanomaterials.

## 7 Design and Optimization of Artificial Bio-Inspired Photonic Surfaces

**Section Authors**: Susete N. Fernandes (NOVA University Lisbon), Savvas G. Chalkidis (National and Kapodistrian University of Athens), Georgios C. Vougioukalakis (National and Kapodistrian University of Athens), and Maria Helena Godinho (NOVA University Lisbon)

The design and optimization of artificial bio-inspired photonic surfaces is an emerging field combining principles of photonics, materials science, and biology. These surfaces are engineered to mimic the optical properties of natural materials, such as peacock feathers, butterfly wings, iridescent fruits, petals of plants, or the cuticle of some beetles. Photonic structures can be applied to solar cells, optical sensors, and displays. In addition, cellulose liquid crystals can produce up-and-coming and environmentally friendly photonic devices.^134^

**Fig. 3:**
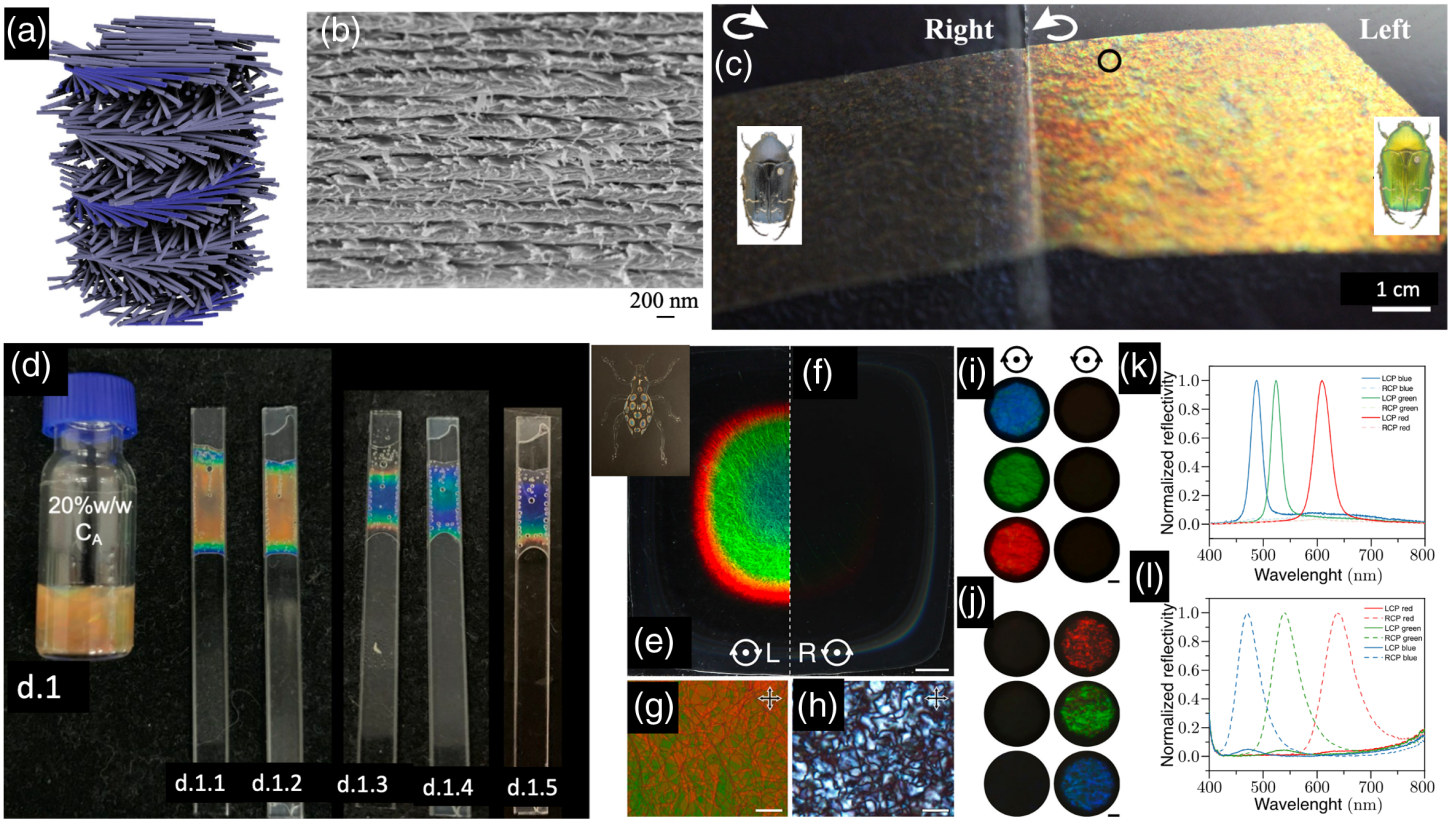
Photonic structures from cellulose systems. (a) Schematic representation of cellulose nanocrystals (CNCs) self-assembled photonic structures. Adapted from Ref. 135 CC-BY 4.0. (b) Cross-section, seen by scanning electronic microscopy (SEM) of the solid iridescent film (in panel c) self-assembled from cellulose nanocrystals in water mimicking the cuticle of Cetonia aurata (inset in panel c). Reproduced from Ref. 136 with permission; © 2016 Wiley-VCH Verlag GmbH & Co. (c) Photographs of beetles reproduced from Ref. 137 with permission; © 2018 Elsevier. (d)–(l) Lyotropic solutions of cellulose derivatives displaying structural colors that change in time (from d.1.1 to d.1.5) and, in thin layers, simulating the sequence of colors^135^ found in the cuticle spots of *Pachyrrhynchus congestus pavonius* (inset in panel e; adapted from Ref. 138 CC-BY 4.0). (d) Reproduced from Ref. 138 with permission from the Royal Society of Chemistry. (e)–(m) Reproduced from Ref. 135, CC-BY 4.0.

Cellulose liquid crystals are biodegradable, renewable, and nontoxic materials that have emerged as a promising alternative to traditional photonic materials. It has been demonstrated that cellulose liquid crystals can produce photonic iridescent films mimicking the cuticle of some beetles, for example, the *Cetonia cerieogram* (Fig. 3), a desirable property for anti-counterfeiting applications. In addition, the films prepared from precursor suspensions of cellulose nanocrystals (CNCs) in water can be combined with other materials, such as low molecular weight nematic liquid crystals, to create tunable photonic structures sensitive to electric fields and temperature.^136^

The chemical functionalization of cellulose-based materials allows for manipulating their properties, leading to a wide variety of applications.^139^ Common modification strategies for CNCs include oxidation by treatment with oxidizing agents such as TEMPO, esterification using acyl halides, O-silylation with chlorosilanes, and etherification using epoxides, as well as functionalization via “Click” Chemistry and nanoparticles incorporation.^140^^–^^142^ Functionalized CNCs have successfully prepared conductive materials, drug-delivery carriers, biocompatible antibacterial agents, temperature-responsive cell encapsulation nano fibrillar hydrogels, and catalytically active surfaces.^141^^,^^143^^–^^147^ Functionalized CNCs have likewise been applied in photonics.^148^ These materials have shown interesting magnetic and optical properties by stabilizing metal nanoparticles and tunable iridescent capabilities by co-assembling zwitterionic surfactants and photoactive polymers onto ${{\mathrm{SO}}_{3}}^{-}$-functionalized CNCs.^149^^–^^151^

Furthermore, the introduction of amino-fluorescent dyes on the CNC structure by acid-amine coupling has been used to prepare bioimaging agents and multi-stimuli fluorescent nanocrystals with responsiveness to solvent polarity, pH, heat, and light.^152^^–^^154^ However, the undisputed potential of the functionalized CNCs is only limited by the available chemical transformation approaches currently employed for their preparation. The chemical optimization of artificial bio-inspired photonic surfaces is still in its early stages, and researchers are actively exploring new materials and fabrication techniques.

### 7.1 Current and Future Challenges

Artificial, bio-inspired photonic surface design and optimization face several challenges that must be addressed for its continued growth and development. One of the primary challenges is the development of scalable fabrication techniques enabling the production of large-scale photonic structures. Currently, most fabrication techniques need to be improved in their ability to produce large-area photonic structures, which limits the practical applications of these materials. Addressing this challenge will require developing new and innovative fabrication techniques that can produce large-area photonic structures cost-effectively and efficiently. Another challenge in the field is the development of stable and durable photonic structures. Many, such as those produced from CNCs, are sensitive to humidity, and other environmental factors, which can impact their optical properties. In addition, CNC photonic structures are fragile and can be damaged easily, which limits their practical applications. Therefore, developing stable and durable photonic structures that withstand various environmental conditions is critical for their practical use in various applications.^155^ The development of innovative modification strategies by focusing on new chemical functionalization that is efficient under mild conditions and with limited waste and byproduct formation is essential for the future growth of the field. Furthermore, the field must also address the challenge of achieving precise control over the optical properties of photonic structures. Currently, most photonic structures produced from cellulose-based materials are limited in their ability to have specific colors and visual effects, thus limiting their practical applications. Therefore, developing photonic structures with precise control over their optical properties, color, and intensity is critical for their use in various applications, including optical sensors and displays. In the future, the field of design and optimization of artificial bio-inspired photonic surfaces will face new challenges as it expands into new areas of research and applications. One such challenge will be the development of photonic structures that can be tuned on demand to respond to changes in temperature or humidity. Developing materials that can sense and respond to changes in their environment has broad applications in sensing and monitoring.^156^ Another challenge will be integrating photonic structures into existing technologies, including electronics and textiles, which will be critical for their practical use in various applications. Overall, the field of design and optimization of artificial bio-inspired photonic surfaces faces several current and future challenges. Addressing these challenges will require collaboration between researchers in various fields, including photonics, materials science, and biology, and developing new and innovative materials and fabrication techniques.^157^ By doing so, researchers in the area have the potential to enable new technologies and applications that are currently not possible.

### 7.2 Advances in Science and Technology to Meet Challenges

**Fig. 4:**
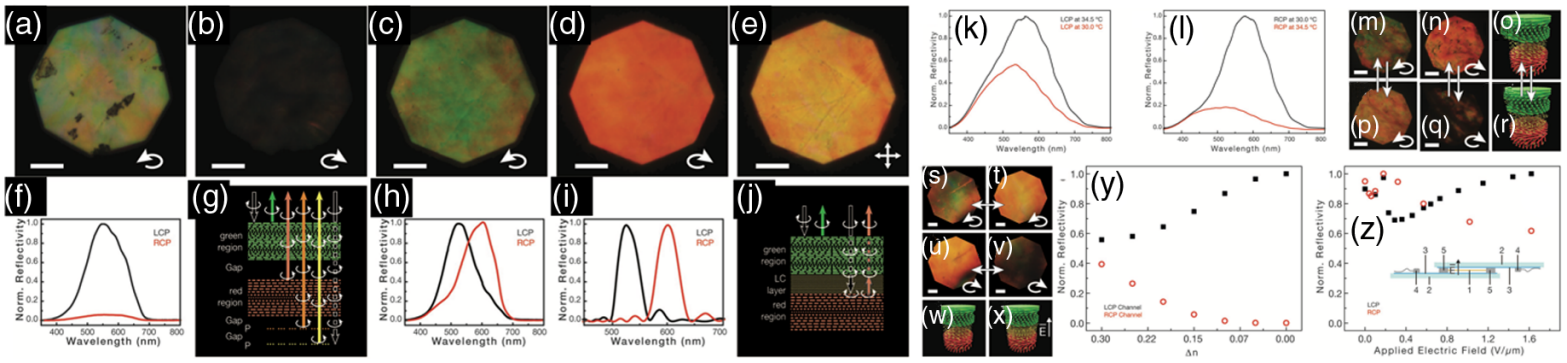
Cellulose nanocrystals (CNCs)/nematic films. (a and b) Initial cellulose helicoidal iridescent film with response in the left-handed channel. (c–e) Cellulose film impregnated with a nematic liquid crystal layer. (f and g) Reflection spectra and a schematic representation of the CNC film functioning. (h–j) Reflection spectra and a schematic representation of the CNC/nematic films functioning. (k–r) CNCs/nematic sensor response to temperature. (s–z) CNCs/nematic sensor response to an electric field. Reproduced from Ref. 136 with permission of John Wiley and Sons, Copyright 2016 Wiley-VCH Verlag GmbH & Co. KGaA, Weinheim.

Advances in science and technology have enabled researchers to overcome some of the challenges associated with designing and optimizing artificial bio-inspired photonic surfaces, particularly in cellulose-based materials. As a result, cellulose liquid crystals have emerged as a promising alternative to traditional photonic materials, offering several advantages in terms of sustainability and performance. Recent advances in the fabrication of cellulose-based materials have enabled researchers to produce helicoidal photonic structures with tunable optical properties [Figs. 4(a) and 4(b)]. For example, researchers have used the self-assembly of cellulose nanocrystals to develop photonic structures with bright iridescence and structural color [see Figs. 4(c)]. As a result, these materials have potential applications in anti-counterfeiting, optical, and display applications. Furthermore, advances in nanofabrication techniques have enabled the production of large-area, uniform photonic structures from cellulose-based materials.^140^ This has been achieved by combining cellulose with other materials, such as polystyrene, to produce photonic structures with high optical quality and durability.^139^^,^^140^ These structures have potential applications in various fields, including solar cells and flexible electronics. Studying photonic cellulose out-of-equilibrium systems opens a new field in bio-inspired surfaces. These surfaces represent functional, active materials that can adapt and mimic photonic sequences of colors existing in plants and animals (for example, Fig. 3 inset in panel e.) as they grow and develop [see Figs. 4(d)–4(m)].^135^^,^^138^ Moreover, advances in understanding the optical properties of cellulose-based materials have enabled optimizing the design of photonic structures, thus achieving specific optical effects. For example, it was demonstrated that the ability to control the right and left-handed circular polarized light of films prepared from CNCs/liquid crystal composites by applying an electric field or by changing the temperature,^136^ by altering the orientation of the LC molecules, or by inducing a phase transition of the LC. This level of control over the optical properties of photonic cellulose-based structures has shown that applications devoted to optics and displays are possible (Fig. 3). In addition, advances in the characterization of cellulose-based materials have enabled researchers to understand their physical properties, such as their mechanical strength and thermal stability.^158^ This knowledge has enabled the development of new fabrication techniques that can produce large-area photonic structures with high optical quality and durability. Furthermore, the biodegradability and non-toxicity of cellulose-based materials offer an advantage over traditional photonic materials, making them more environmentally friendly and sustainable. Progress in science and technology has enabled researchers to overcome many challenges in designing and optimizing artificial bio-inspired photonic surfaces. In particular, the development of cellulose-based materials has shown great promise in sustainability, performance, and tunability. These advances have potential applications in various fields, including sensing, energy, and displays. Continued research in this area is expected to result in further advances in the design and optimization of photonic structures, enabling new technologies and applications.^137^

In conclusion, the design and optimization of artificial bio-inspired photonic surfaces hold great potential for various applications in sensing, imaging, and energy harvesting. One of the critical components in such bio-inspired surfaces is cellulose, a versatile and abundant material that can be used in various forms, such as nanofibrils, nanocrystals, and microfibers.^159^ Using cellulose in artificial bio-inspired photonic surfaces offers several advantages, including high strength, biodegradability, and low toxicity. Moreover, it can be processed using cost-effective and scalable methods such as solution casting, spin coating, and inkjet printing. Cellulose-based bio-inspired photonic surfaces can be optimized by tuning the surface morphology, chemical composition, and processing parameters. Such optimization can enhance optical properties, including high reflectivity, broadband absorption, and directional scattering. In a nutshell, the use of cellulose in artificial bio-inspired photonic surfaces has immense potential as an active functional material. Further research in this field can develop novel materials with unique optical properties for various applications.

**Acknowledgments**

The authors acknowledge support from Action European Topology Interdisciplinary Action (Grant No. PhoBioS CA21159) and FCT-Fundação para a Ciência e a Tecnologia, I.P., in the scope of the projects LA/P/0037/2020, UIDP/50025/2020, and UIDB/50025/2020 of the Associate Laboratory Institute of Nanostructures, Nanomodelling, and Nanofabrication-i3N.

## 8 Cell walls of diatom microalgae with photonic properties: potential for biomimetic manipulation through genomics and synthetic biology

**Section Author**: Atle M. Bones (Norwegian University of Science and Technology)

### 8.1 Summary

Diatom silica frustules can handle optical radiation through diffractive, refractive, and wave-guiding processes. Nature has evolved specialized proteins and polyamines to control the biomineralization of silica in diatoms producing a glass wall (frustule) around the cells. The molecular mechanisms underlying frustule formation in diatoms are offering insights and could inspire biomimetic approaches for materials synthesis and engineering of photonic structures. Omics technologies open for studies of the underlying biological mechanisms and regulation, revealing key molecular players and their function, and directing the route to optimize biological processes for applications. Such identification of targets by genomics, transcriptomics, proteomics, metabolomics, and other omics opens possibilities to establish a synthetic biology module system with genetic elements that will allow us to influence and manufacture novel frustule forms and structures. This biomimetic strategy could produce an array of nanomaterials for a range of applications.

#### 8.1.1 Diatom biomineralization and photonic properties

**Fig. 5:**
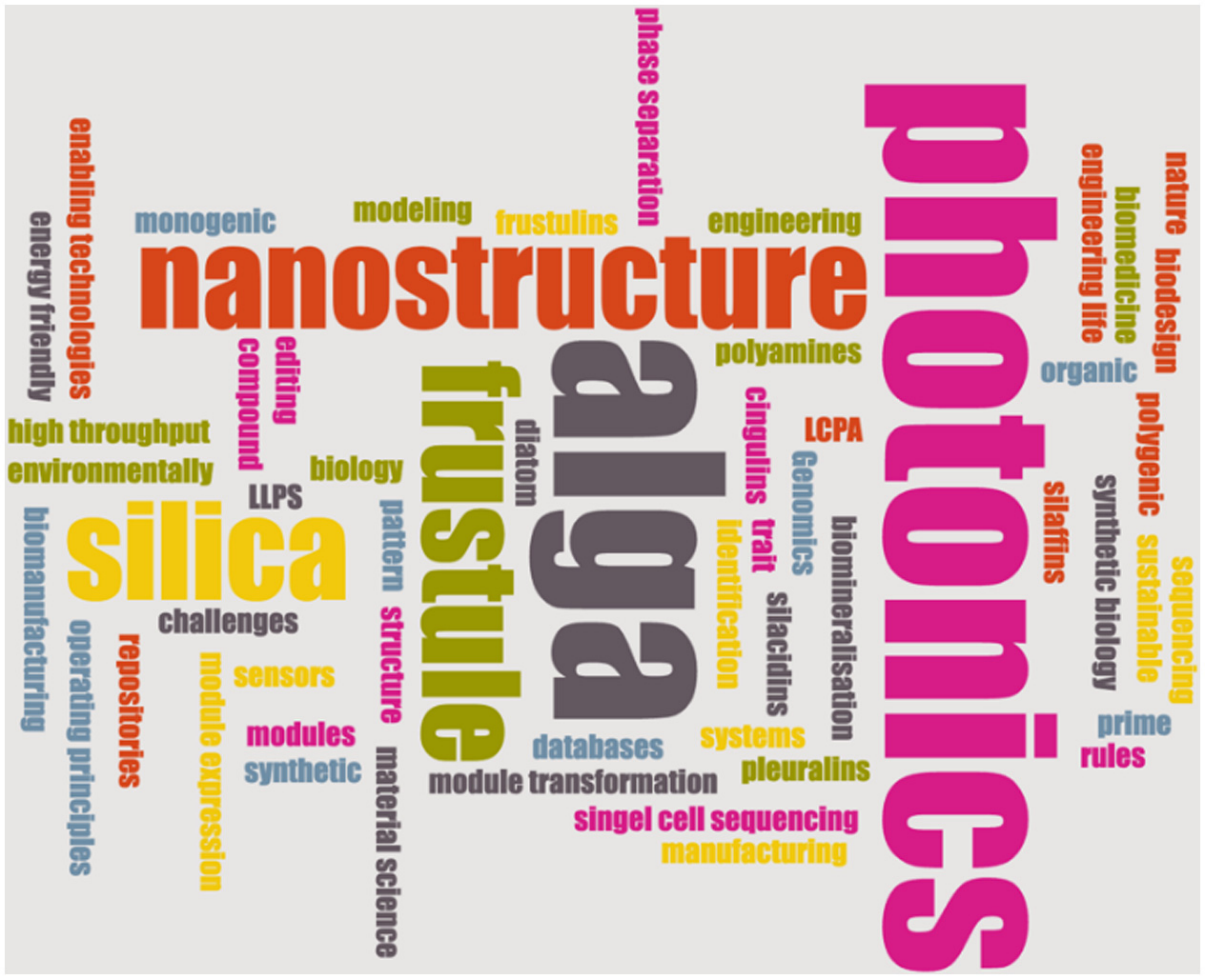
Word cloud summary indicating some issues to address. Created with WordClouds.^160^

**Fig. 6:**
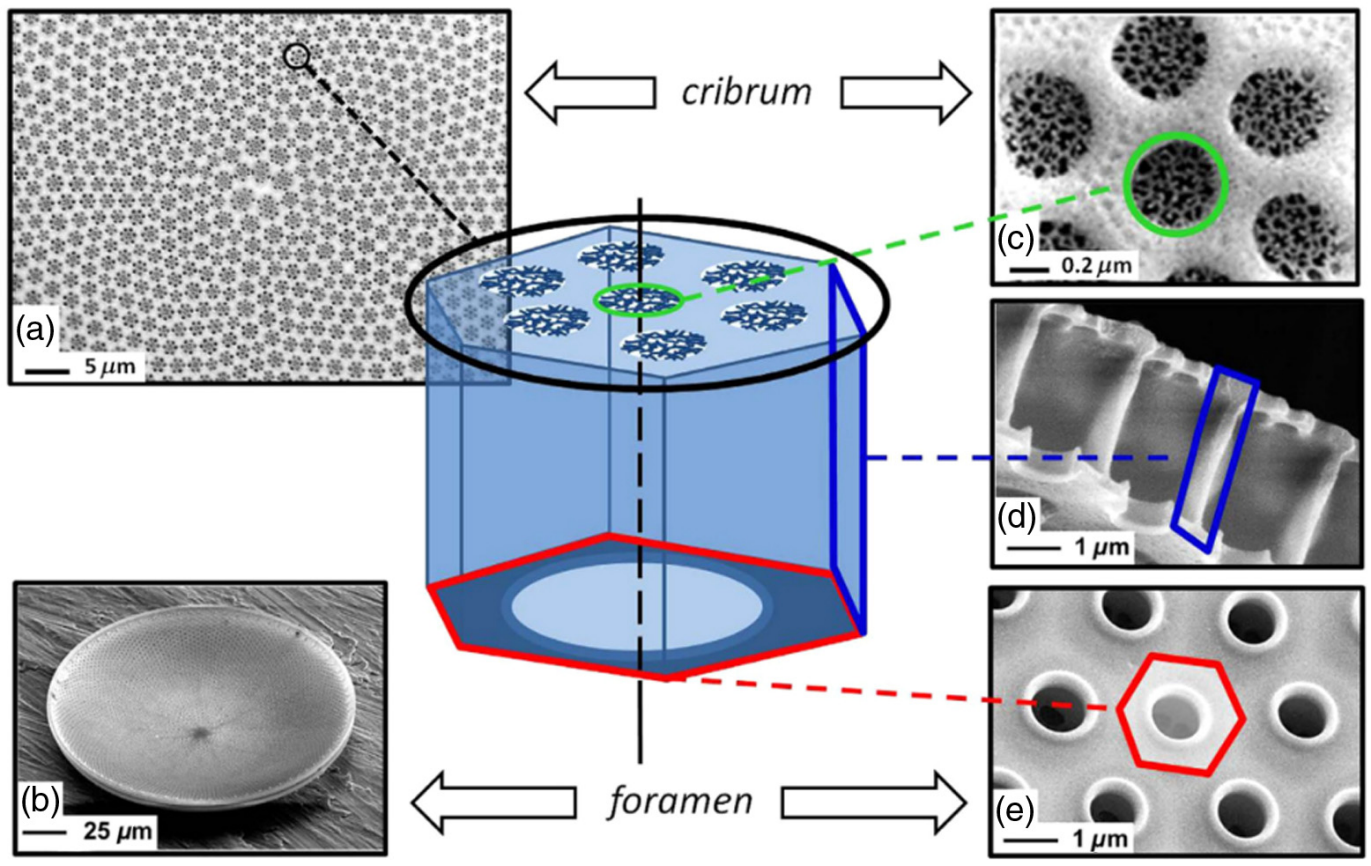
Generalized structure of a diatom (*Cosinodiscus sp*) bio-silica frustule valve and details imaged with SEM. Reproduced with permission from Ref. 161.

Biomineralization refers to the process by which living organisms produce minerals, often in the form of crystals. This process is controlled by biological molecules such as proteins, lipids, and polysaccharides, which act as templates, scaffolds, or regulators for mineral deposition. Biominerals produced by organisms have diverse functions, including structural support, protection against environmental conditions and predators and storage of essential ions. Nature produces mineralized structures of surprisingly high precision and complexity at macro- to nano- scale levels. One example is frustules (Figs. 5 and 6), the nanopatterned silica cell walls of diatoms with photonic properties.^161^^–^^166^ Diatoms are unicellular algae in the millimeter range size (∼2−200  mm) and have an important role in biogeochemical cycling of silicon and other elements such as nitrogen, phosphorus, carbon, and iron in addition to their prime role as one of the major primary producers of the oceans. In addition this biomineralization is done at ocean ambient temperature in an environmentally friendly process at high-speed and low-energy consumption. Unicellular diatoms produce their silica walls inside the cell. An array of patterns can be produced by different algae species ranging from simple structures to cell walls with layers and extremely well-organized patterning. One example from a frustule valve of a large *Coscinodiscus* alga is shown in Fig. 6. The processes behind the biomineralization are only partially understood and involve interactions between silica, proteins/peptides, polyamines, and a series of modifications affecting the properties of the major components. The bio-manufacturing of the silica is mainly done in a cellular structure named silica deposition vesicles (SDV) or originally silica transport vesicles (STV).^167^ There are also results indicating that some silica structures are assembled outside the cell and not in the typical SDVs.^168^ To control biomineralization of silica in diatoms, specialized proteins are produced, which interact with the silica species.^169^^,^^170^ The process of silica mineralization has been studied for decades.^171^^,^^172^ Sumper and co-workers found evidence for a role for long-chain polyamines (up to 20 repeated units) in the control of silica morphology.^169^^–^^171^^,^^173^^,^^174^ In an early experiment, it was shown that specific long-chain polyamines together with silica-precipitating proteins named silaffins induced silica precipitation from a silicic acid solution.^169^ A synergetic action of polyamine specimen and silica-precipitating proteins was observed, which strongly indicates that different species of diatoms are equipped with a subset of specific polyamines and associated proteins controlling their silica patterning.^169^ A liquid–liquid phase separation model for nanopatterning of diatom silica was suggested,^171^ indicating a concentration-dependent pore size formation, which partly could explain the silica patterns observed between diatom species.

#### 8.1.2 Identification of targets for bioengineering of photonic silica

Omics- and especially functional genomics opens possibilities for exploitation of the processes behind the mineralization of the diatom cell wall and other (mineralized) biostructures. Pre-omics age was dominated by methods that specifically targeted one compound or group of compounds at a time. This could be a protein, a gene, or some metabolite. Considering that there are thousands of genes and hundreds of thousands of products in a cell at any time, this only targets a very small fraction of the components present, which limits the possibility to predict co-regulation and pathways that are operative and thereby mechanisms behind a process. Genomics, proteomics, metabolomics, and other omics-related methods have replaced many of the very time-consuming and challenging reductionistic techniques in the search for the function of components and associated mechanisms. Characterization of the function of a component, such as a protein, could require several years of research and was, in many cases, incomplete because the studies often only focused on one component at a time and were unable to provide data on the bigger (interaction) picture. Genomics is much more useful for exploring the unknown. The potential and power of the novel omics methods were clearly shown during the COVID-19 pandemic. There would be no way to identify, characterize, and produce vaccines against the unknown pathogen in a timely manner without the omics and nucleic acid synthesis technologies developed over the last few decades. Identification of the genetics behind silica precipitation and patterning is under investigation and high throughput methods such as transcriptome profiling and proteomics have revealed at least some of the processes and molecular systems involved.^170^^,^^175^ Brembu et al.^170^ performed a transcriptome analysis to identify signature genes and their corresponding proteins involved in responses to silicon replete or deplete conditions. Hypothesis being that candidate genes involved in the biosilicification could be identified among the differentially regulated and co-regulated genes and that identified candidates could serve as targets for functional analyses of their role in silica cell wall production through genome editing.^176^^–^^178^ Heintze et al.^175^ used a genomics approach to identify and target a specific family of proteins with ankyrin protein–protein interaction domains. Proteins with ankyrin repeats are thought to have a primary function in mediating protein–protein interactions, serving as scaffolds or adaptors, and they are characterized by repeats of amino acids forming a helix-loop-helix structure folding into a compact solenoid-like domain. By genetic manipulation of the genes encoding the ankyrin motif-containing proteins, they produced knock-out mutants of diatom cells with modified frustule silica nanostructures. The overall structure of the silica cell walls was conserved, but knock-out of each of the three dAnk1-3 genes resulted in pore pattern modifications such as a reduced number of nanopores or a different organization of the nanopores.^175^

#### 8.1.3 Modification and engineering and the potential of synthetic biology

High throughput omics technologies provide a source of comprehensive and massive data revealing sequences, presence (ratios), and conditional levels (of changes) for genes, transcripts, proteins, metabolites, and other molecules. A fundamental part has been the whole genome sequencing of model species and hundreds of other species over the last two to three decades.^179^^,^^180^ This sequence information is utilized in the annotation of gene/gene models, phylogenetic analyses/evolution, for protein identification in proteomics, in predictive modelling by artificial intelligence (AI) models such as Alphafold and more.^181^ In brief, the databases/repositories of DNA sequences, transcript, protein, and metabolite profiles, and libraries/repositories, together with stock centers harboring mutants and genetic constructs/modules are essential for the exploitation and further development of synthetic biology. Data from these resources provide a foundation for a more holistic view of biological activities in given developmental stages and conditions than the traditional reductionist methods. Integration of such datasets makes it possible to gain insight into complex biological systems, networks, and organismal-specific systems distribution.^182^^,^^183^ Understanding of continuous processes and dynamic changes is though hampered by experimental constraints such as the limited number of time-points sampled and observed (cost and time), incomplete annotations, and limitations in methodologies employed. Despite such limitations, comprehensive datasets can be compiled on the biological process of interest, and integration of data from different technology platforms is useful to gain a more holistic understanding of the biological system.

**Fig. 7:**
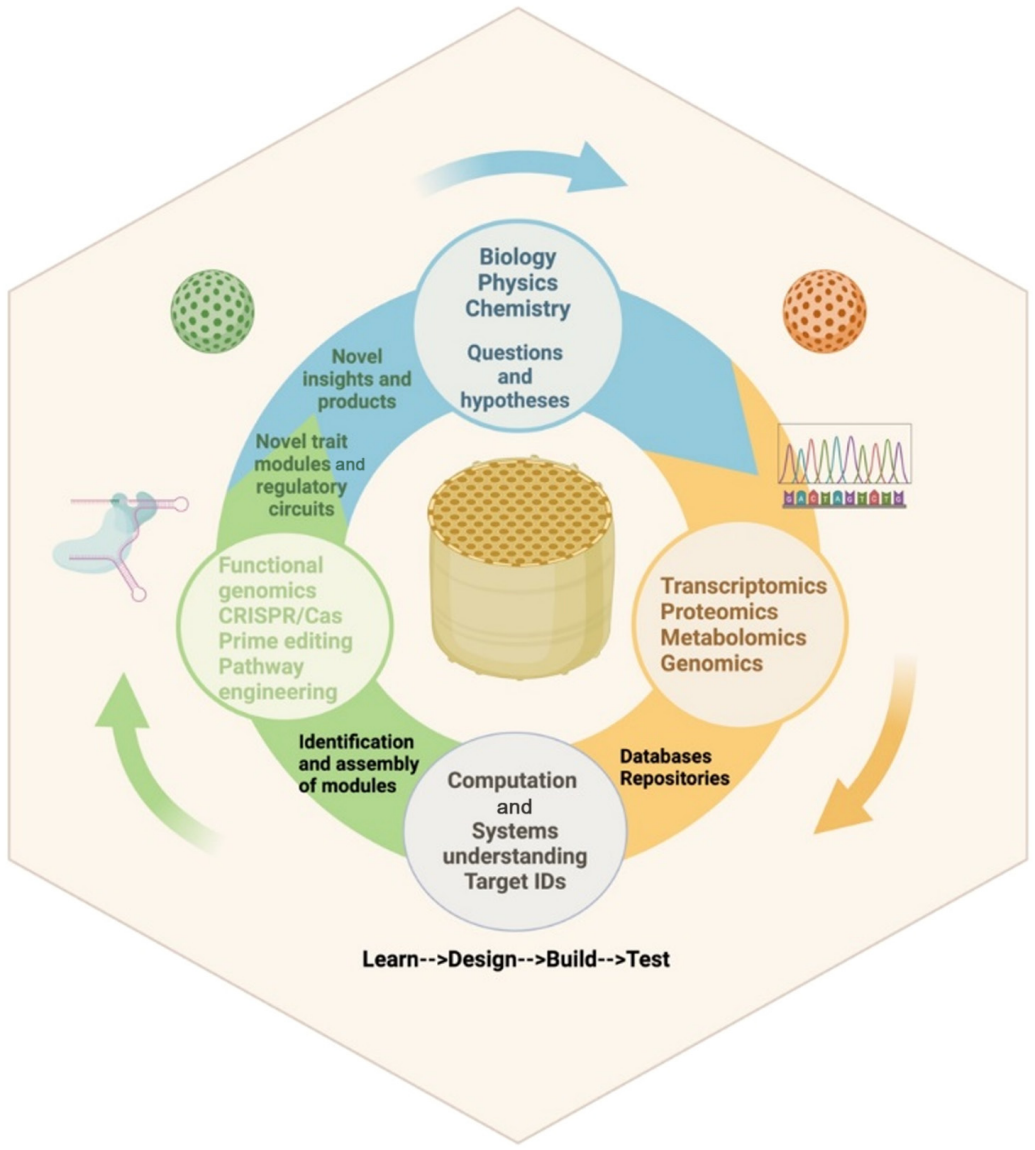
Systems biology approach for identification and design of trait encoding modules (TEMs) Created with BioRender.^187^

Transcriptional, protein, or metabolite profiles will often lead to identification of (novel) candidate gene/proteins/metabolites and/or biosynthetic pathways or regulatory systems involved. Thereby producing, e.g., gene candidates for cellular functional studies using knock-out, overexpression, etc., for functional studies. CRISPR/Cas genome editing methods are now widely used for verification of the cellular process of interest and further annotation.^178^^,^^184^^–^^186^ The principles of omics-technologies have not only opened new avenues for studies of fundamental processes of life but also been a game-changer for applied research. Transcriptomics, proteomics, metabolomics, and other omics technologies provide opportunities to study the different parts and processes of a cell (Fig. 7). Instead of specific targeted measurements of single components of the cell/tissue/organism, techniques such as transcriptomics allow an integrated study of all mRNAs, all non-coding RNAs, and small RNAs. Similarly, we can utilize proteomics techniques to get the big picture of peptides/polypeptides/proteins produced/present in a given cell and condition. Together with time series and conditional data, these techniques provide us with a picture of cellular machineries that are active. In addition, this can be analyzed at the single cellular level or even at a cell compartment/structure level. One very prominent example is the technology development of high-throughput sequencing that allows simultaneously analyses of thousands to millions of DNA or RNA molecules. Combined with proteomics and metabolomics, these are powerful methods for target identification and selection of candidate genes/proteins for functional analyses and bioengineering. The principle is often that samples from various time points or conditions are compared, and co-regulated components are clustered or categorized. This is then followed by process/pathway analyses and prediction of controlling elements such as a transcription factor^188^ or an essential enzyme in the pathway.^189^^,^^190^ These studies are often supported by machine learning and other software and open AI tools such as AlphaFold.^181^ In many labs, Alphafold is now routinely used for the prediction of protein structures and support for functional studies. Functional analyses will then reveal whether the component or module has the predicted role. Synthetic biology is a potentially powerful tool for the development of modular trait constructs, the reconstruction of the genetics of an organism or even building novel organisms from a toolbox of elements.^186^^,^^191^^–^^199^ It has already been proven that a self-replicating cell can be produced from an assembly of completely synthetic genetic elements.^191^^,^^192^ It is tempting to expect synthetic biology to expand rapidly with the help of the recently developed genomics methods and the fast-growing field of AI. Many traits are polygenic in nature and established as a sum of influence from multiple genes and regulatory systems. We are now at a stage in science where genetic elements can be combined in modules and exchanged between individuals, species, or organismal types.^197^ It is easy to oversell, but biotechnology and synthetic biology have enormous potential. AI is already used in, e.g., text generation. The same principles might apply to rewriting the code of life: genetics. Fueled with genetic information from thousands of species, AI principles might be used to design molecules, traits, and processes of hitherto unknown functionalities. Principles that, e.g., can be used to design novel biomineralized materials in a sustainable process and mass cultivate^200^ at ambient conditions for a range of applications.

#### 8.1.4 Conclusion

Silica frustules are used as an example of a biomineralization process, where there are possibilities for biomimetics to manufacture structures with photonic properties. Progress has been made on the mechanisms of diatom frustule formation. However, there is still a way to go before a systems biology understanding of the frustule biomineralization process is established, and the frustules can be manipulated or modeled to fully exploit their ability to handle optical radiation. This requires control of the individual components as well as all the interactions and conditions throughout the process. That said, the first gene-edited cells with modified nanopore structures have been made based on genomics. In addition the potential to manipulate pore structure and properties has been proven by simple single-gene manipulations. Silica frustule mineralization is encoded and controlled by multiple genes/proteins and mechanisms. This may complicate control of the silica formation, but likely also open a wider array of configurations possible, guided by the power of synthetic biology.

## 9 Optical Tissue Phantoms for Biomedical Applications

**Section Authors**: Maciej S. Wróbel (Gdańsk University of Technology) and Katarzyna Karpienko (Gdańsk University of Technology)

Advances in optical technologies, including materials, devices, and data processing algorithms in recent years, have enabled optical diagnostic tools to be translated into clinics, and some of them are on the way to being adapted on a larger scale and eventually reaching clinical practice. However, one issue, among others, still remains slightly lacking behind in terms of standardization^201^ and practical utilization, and those are optical tissue phantoms. Being mostly uninteresting slabs of scattering material, their purpose is however quite important because they are key to ensuring the proper calibration and validation of these emerging devices. Yet, even though their importance is well known and many different materials have been used, there are still challenges regarding their widespread application and standardization.^201^^–^^208^

### 9.1 Current and Future Challenges

Phantoms in a medical setting are objects whose purpose is to simulate some of the properties of real life objects, or mimic them as a whole, which can range anywhere from singular tissues, such as learning to perform skin stiches, or organs for performing procedures such as gastro- or colonoscopy, up to whole systems such cardiovascular-respiratory system, or whole body parts—prosthetics, or organs, mainly for training purpose of medical procedures.

In the case of optical tissue phantoms, they serve mostly a similar purpose, but for training and validation of light-based diagnostic and treatment tools, rather than the personnel. In addition, the phantom’s purpose may be a bit different, that is when their properties are made to mimic, or match, only certain parameters. Then, they serve the purpose of ensuring proper readout of specific sensors or larger medical devices. Thus, they find utilization as calibration standards. In this case, they need to be very precisely measured using other established measurement methods. Another purpose is personalized medicine, where a phantom can be used for calibration specific to the intended user, such as precise dosimetry, which is often the case in, e.g., radiative medicine, or surgical imaging.^202^

In the case of optical methods, we can group optical tissue phantoms into categories dependent on their combination with the basic physical or chemical phenomena on which those methods rely:

• **Turbidity**—simulating mostly a single-wavelength scattering and absorption. Usually, the most generic type, useful for in-and-out light transport, dosimetry, or light delivery for surgery or cosmetic medicine.
• **Spectroscopy**—these phantoms focus on the absorption profile along the wavelength, for example, to mimic blood perfusion in tissues, and are most commonly used in UV-VIS-NIR spectroscopy, hyperspectral imaging, or even in fluorescence-based methods.
• **Emission**—these phantoms are used in methods such as the fluorescence, although they can also be used in other methods where the phantoms themselves are the source of radiation.
• **Chemistry**—specific chemical substances are the target of some methods such as Raman spectroscopy or IR spectroscopy, where the specific molecular bonds are looked for; thus, these phantoms should contain the same or similar chemistry.
• **Mechanical/acoustics**—phantoms often also mimic the physical properties such as the elasticity or sound wave speed, which is used in opto-acoustic and related methods.
• **Thermal**—in cases of photo-thermal effects, phantoms should also have a similar thermal response to laser heating, which is often used in phantoms with a large water content.^209^^–^^214^

Of course, this is only a rough division, and many phantoms often are made with properties that span multiple categories. More modern phantoms combine multiple methods and thus require greater consideration in the choice of materials or combinations thereof. From this, and their intended purpose, we can establish some key parameters that a “perfect” phantom should have, these include but are not limited to:

• Reliability of parameters,
• Stability over time,
• Low cost of fabrication,
• Ease of fabrication,
• Ability to combine multiple functions,
• Refractive index similar to tissues,
• Absorption and scattering similar to intended tissues,
• Similar physical dimensions,
• Ability to introduce additional features, lesions, and vessels,

Although these were well-known properties of phantoms for many years, these challenges are unresolved due to conflicting parameters of materials, or complex structures, the need for specific chemical substances, and last but not least, the practical parameters such as fabrication ease and costs.

### 9.2 Advances in Science and Technology to Meet New Challenges

Most optics-focused tissue phantoms are created to match only the turbidity. That is absorption (absorption coefficient $\mu_{a}$) and scattering of light, namely the scattering coefficient $\mu_{s}$, or more often the $\mu_{s}^{\prime }$—reduced scattering coefficient. The challenge of mimicking properties accurately here is significant, because the $\mu_{s}^{\prime }$ does not mimic properly the anisotropy of light scattering (directionality of scattering, often noted as the g-factor), which may introduce some problems with directional light measurements, off-axis from the incident source, or introduce errors when calibrating diffuse optic–based methods. Another problem is the refractive index mismatch, because it will generate unwanted Fresnel reflections, which may contribute to excess light being detected or more light being lost, depending on the measurement geometry.

Measurement of optical properties of tissues,^215^^–^^218^ and phantoms alike, is a challenging process as it requires specific instrumentation and computation, the most accurate being the IAD method.^219^^–^^221^ Development here would significantly reduce the problem of phantom cost because measurements are arduous and problematic.

Most common materials^222^^–^^226^ used in phantom fabrication are made of a possibly optically transparent base material, which forms the bulk of the phantoms. These include water, agar, or a multitude of polymers, such as PVCP, PDMS,^227^^–^^229^ etc. Within the base, it is introduced a scattering medium, most often scattering particles known for use in paints, such as metallic nanoparticles alumina ${\mathrm{Al}}_{2}O_{3}$, titanium dioxide ${\mathrm{TiO}}_{2}$, zinc oxide ZnO, barium sulfate ${\mathrm{BaSO}}_{4}$, etc., silica or polymer microspheres, or even without nanoparticles,^230^^,^^231^ such as using materials that scatter on its own, such as the most well-known intralipid.^232^^,^^233^ Challenges here remain in finding materials or a combination thereof that are generally more accurately mimicking scattering, while having almost no absorption or absorption spectral profile beyond the intended purpose wavelength range.

Another challenge besides the simple turbidity is accurate mimicking of the spectral profile of absorption within phantoms. This is very important and has been extensively used to mimic blood parameters such as perfusion and oxygenation,^228^^,^^229^^,^^234^^–^^237^ or lesion discoloration. These phantoms’ properties must follow the dependence on the wavelength of light, thus are often created either for only a specific wavelength, as is often the case in previously mentioned phantoms, but it is rarely enough with spectral methods. Alternatively, more sophisticated phantoms of this type additionally strive to mimic the spectral characteristics by matching dyes and pigments’ spectral profiles or their mixtures to simulate for example the specific bands of melanin, hemoglobin,^228^^,^^238^^–^^240^ and also other applications such as tattoo inks safety.^241^

Moreover, a common application is mimicking different structures,^242^^,^^243^ similar to pathological lesions, such as dermal changes in melanoma. Others are inclusions, such as in-depth capillaries or vasculature.^234^^,^^235^^,^^244^^–^^247^ These phantoms could be utilized in diffuse spectral measurements,^211^^,^^216^^,^^221^^,^^225^^,^^235^^,^^240^^,^^248^^,^^249^ in optical imaging, or even in hyperspectral imaging.^236^^,^^250^^,^^251^ With vasculature comes the introduction of flow-channels, which can be used for fluid (blood) flow, such as in Doppler methods.

Spectral characteristics can also be mimicked for light emission, such as fluorescence.^252^^–^^254^ Fluorescence is often a quality of contrast agents to visualize specific morphology and differentiate it from other tissues. These phantoms utilize actual fluorescent dyes and are often used for fluorescence imaging or fluorescence-based lesion detection for example in surgical guidance.^207^ Other options include the use of external light sources introduced within the phantoms, to mimic the emission of light.

Another spectral-based phantom applications are its chemical properties, mainly used in spectroscopic methods, and especially important in vibrational spectroscopic methods, mostly Raman spectroscopy, but also to some extent IR spectroscopy. Typical UV-VIS-NIR methods were described earlier as they match absorption bands of electronic transitions, which are broader and less complex. Actual chemistry or very similar materials should be used to provide matching emission/absorption of vibrational bands for Raman/IR. These can be utilized for the detection and recognition of specific disease markers, monitoring of chemical parameters of tissues, drug monitoring, etc., especially taking into consideration the possibility of quantification of these substances.^255^^–^^259^

Emerging methods utilize phantoms, which focus on inclusions of additives into phantoms for specific cases, i.e., for plasmonic-based sensing or plasmonic photodynamic therapy. Various nanoparticles, most often gold nanospheres, nanorods, etc., are also mixed in. One of the novel methods for phantoms fabrication^242^^,^^260^ is 3D printing^261^^–^^264^ of phantom layers^265^ and multiple shapes, with also printed high-resolution cell structures.^266^

A challenging aspect in emerging phantom designs is the combination of other modalities besides optical.^255^^,^^267^^,^^268^ These are often the ultrasound, where the mechanical wave travel velocity is the most important parameter, which are used in photoacoustic methods as well as combined optical imaging and USG devices.^255^^,^^269^^,^^270^ Besides mechanical properties, magnetic properties can also be mimicked by embedding magnetic materials, such as nanoparticles, along with the optical part of the phantom.

In conclusion, the optical properties of tissues are very complex when thinking beyond single wavelengths and bulk tissues. Especially taking into consideration the morphology of tissues, the phantoms are becoming more and more complex, including multi-layers. Lesions, inclusions, vessels, and other additives. They often also need to mimic specific shapes of bulk tissues or internal organs. The further development of tissue phantoms is guided by the specific needs of the methods it needs to help calibrate; thus, one can assume phantom standardization in the future coming in many different types, or sets of phantoms for specific purpose, but most likely the low-cost and ease of fabrication will not be the driving factors, as reliability and stability of parameters in a clinical practice setting is always preferred.

**Acknowledgments**

This research received financial support from the project “Excellence Initiative - Research University” (Grant No. DEC-3//2021/IDUB/II.2/Scandium), Argentum Triggering Research (Grant No. 30/1/2022/IDUB/I3b/Ag), and the DS funds of the Faculty of Electronics, Telecommunications and Informatics of the Gdańsk University of Technology.

## 10 Optical Detection of Biomarkers in Wastewater using Spectroscopic and Machine-Learning Methods

**Section Authors**: Adam Władziński (Gdańsk University of Technology and ECO CHAIN), Marta Władzińska (ECO CHAIN), and Małgorzata Szczerska (Gdańsk University of Technology)

The analysis of wastewater using optical techniques—such as UV-Vis spectroscopy, fiber-optic interferometry, and Raman spectroscopy—encounters significant challenges due to the highly complex biochemical composition, as summarized in Table 1.^271^ Wastewater contains a diverse mixture of organic and inorganic compounds, including microorganisms, algae, fibers, salts, oils, and various contaminants,^272^^–^^274^ all of which generate overlapping and difficult-to-interpret optical signals. As a result, accurate measurements in such heterogeneous environments remain technically demanding and methodologically complex.^273^

In response to these challenges, we develop wastewater models, termed wastewater optical phantoms, optically and chemically equivalent media explicitly designed to replicate the complexity and optical characteristics of real wastewater.^275^ These phantoms accurately mimic the absorption, scattering, and chemical composition observed in authentic wastewater samples, providing stable and reproducible experimental conditions. Such optically equivalent phantoms facilitate systematic studies and reliable validation of novel sensors and analytical algorithms, overcoming the methodological variability inherent in real-world environmental matrices.^274^^,^^276^

**Table 1:** Comparison of real wastewater samples and wastewater phantoms Wastewater phantoms provide reproducible, controllable, and standardized conditions for biomarker studies and diagnostic method validation.

Feature	Real wastewater samples	Wastewater phantoms
Chemical composition	Highly complex, heterogeneous, variable	Precisely defined, controlled, reproducible
Optical properties	Variable absorption and scattering signals	Stable, calibrated optical characteristics
Experimental repeatability	Low, dependent on external conditions	High, consistent across samples
Applicability in diagnostics	Direct, but technically challenging	Indirect, but methodologically advantageous
Suitability for machine learning	Challenging due to variability	Excellent due to standardized conditions

Despite increasing interest in optical sensing and machine learning for biosensing, there is still a lack of robust studies involving real wastewater matrices. Most methods developed so far have been validated using simplified media, such as phosphate-buffered saline (PBS) or synthetic urine, rather than actual wastewater samples. This limits their real-world applicability and makes cross-validation with realistic systems difficult.

Building on these wastewater models, we develop refined artificial wastewater phantoms that closely mimic the optical properties—specifically, absorption and scattering—as well as the chemical composition of real wastewater samples. The objective is to produce stable and reproducible test media that allow for controlled and systematic investigations of specific biomarkers of interest.^277^

Wastewater phantoms enable systematic and precise investigations of the presence of specific biomarkers. By adjusting the composition of the phantoms (such as drying or dilution), it is possible to isolate and quantify the optical contribution of individual biomarkers, thereby enabling the determination of optimal detection parameters under controlled conditions.^278^

The application of machine learning techniques—such as Principal Component Analysis (PCA), Support Vector Machines (SVM), and Random Forest—significantly enhances the interpretability and diagnostic value of optical measurements.^272^^,^^279^ These algorithms enable more accurate differentiation of subtle signal variations associated with the presence of specific biomarkers in wastewater phantoms.^272^

The development and use of realistic wastewater phantoms therefore represents a critical step toward bridging the gap between laboratory scale proof-of-concept studies and the deployment of reliable diagnostic systems in real-world environmental monitoring applications.

Challenges in the further development of wastewater phantoms are related to the ongoing need to enhance their realism and improve the accuracy with which they replicate the complex biological and chemical properties of actual wastewater samples.^274^

Optical measurement of wastewater is confronted with multiple obstacles that limit the precision of analysis and the reliability of monitoring results. Foremost among these is the high complexity of the wastewater matrix. Each sample is compositionally unique and subject to seasonal variation, differing significantly depending on the source, season when the sample was collected, and local environmental conditions. One of the critical factors affecting spectroscopic results is the water content, which strongly absorbs light in the near-infrared region—particularly at wavelengths of 785 nm and 830 nm commonly used in Raman spectroscopy.^273^

Additional environmental variables such as temperature, turbidity, and the presence of interfering chemical substances further degrade measurement quality.^280^ Sensor biofouling and corrosion—exacerbated by microbial activity and the chemically aggressive nature of wastewater—lead to signal loss and increased maintenance requirements.^279^ Moreover, calibration remains a technically demanding task due to the lack of standardization across systems, making inter-laboratory comparisons difficult.^274^ Finally, the high implementation and maintenance costs of optical systems, combined with the shortage of experts in integrating optical methods with machine learning, present a significant barrier to wider adoption.^272^

Advances in both optical technologies and machine learning have provided powerful tools that enable increasingly precise analyses and the development of more realistic models and phantoms.^272^ The expanding capabilities of these technologies allow for the integration of various measurement techniques—including UV-Vis spectroscopy,^276^ Raman spectroscopy, and interferometry—with statistical analysis and machine learning, significantly improving the sensitivity, and specificity of the resulting methods.^278^ Algorithms such as Principal Component Analysis (PCA), Support Vector Machines (SVM), and Random Forests are effective in detecting subtle patterns within the data and minimizing the impact of background noise.^272^ Furthermore, ongoing efforts in data fusion from multiple sources contribute to generating a more comprehensive and reliable representation of sample characteristics.^280^

In the domain of phantom construction, it is now feasible to incorporate a broader range of physicochemical parameters, such as water content, sample complexity, and seasonal variability, thereby improving their consistency with real wastewater samples and enhancing environmental representativeness.^277^ Concurrently, advancements in miniaturization and the decreasing costs of optoelectronic components—including laser diodes and detectors—are paving the way for the development of more affordable and portable measurement systems.^279^ Despite these significant advancements, a pressing need for the creation of open-access datasets and the establishment of standardized measurement protocols remains unchanged. These datasets are essential for ensuring result comparability and improving the validation of machine learning algorithms applied to real-world environmental and biomedical applications.^274^ The refinement of wastewater optical phantoms combined with advanced data analysis methods paves the way for the development of effective environmental monitoring systems.^273^ By enabling controlled manipulation of chemical composition and optical properties, these phantoms facilitate the testing of novel sensors and analytical algorithms in environments that closely mimic real-world conditions while ensuring full repeatability and experimental safety.^277^ Advanced analytical approaches, including machine learning, not only automate the interpretation of measurement data but also enhance sample classification accuracy, biomarker identification, and the detection of atypical contaminants.^272^^,^^280^^,^^281^ Techniques such as PCA, SVM, and Random Forest are particularly suited for processing high-dimensional datasets and adapting analyses to the variable composition of wastewater matrices.^278^

These capabilities enable the creation of integrated diagnostic systems, wherein the sensing module (e.g., a Raman spectrometer) interacts in real time with a data analysis unit.^282^ This integration supports the development of compact, field-deployable “lab-on-a-chip” devices for rapid wastewater quality assessment at treatment plants, monitoring stations, or mobile laboratories. In a broader context, the evolution of wastewater phantoms and modeling approaches, supported by optical and machine learning technologies, holds the potential to standardize diagnostic procedures and ensure comparable results across research centers and geographic regions.^274^^,^^275^^,^^277^^,^^282^ This could contribute to the formation of global wastewater surveillance databases, aiding environmental policy decisions at local, national, and international levels.^280^

Ultimately, such technological advancements not only support environmental protection efforts but also improve public health by enabling the early detection of biological and chemical threats in sewage systems, thereby enhancing emergency response readiness in cases of contamination, infrastructure failure, or outbreak detection via wastewater analysis.^273^

**Acknowledgments**

This research was supported by the Ministry of Science and Higher Education under the project “Nauka dla społecze´nstwa w czasie występowania zagroże´n epidemiologicznych” (Grant No. NdS/551425/2022/2022, 2023?2024) and by Gdańsk University of Technology through the PLUTONIUM project (Grant No. DEC-6/1/2025/IDUB/III.4a/Pu, 2025). Both projects were led by Prof. Małgorzata Szczerska. Additional support was provided by the COST Action No. CA21159.

## 11 Monte Carlo Modeling of Light Interaction with Biological Tissues

**Section Authors**: Tatiana Novikova (École Polytechnique and Florida International University), Jessica C. Ramella-Roman (Florida International University), and Igor Meglinski (University of Oulu and Aston University)

**Fig. 8:**
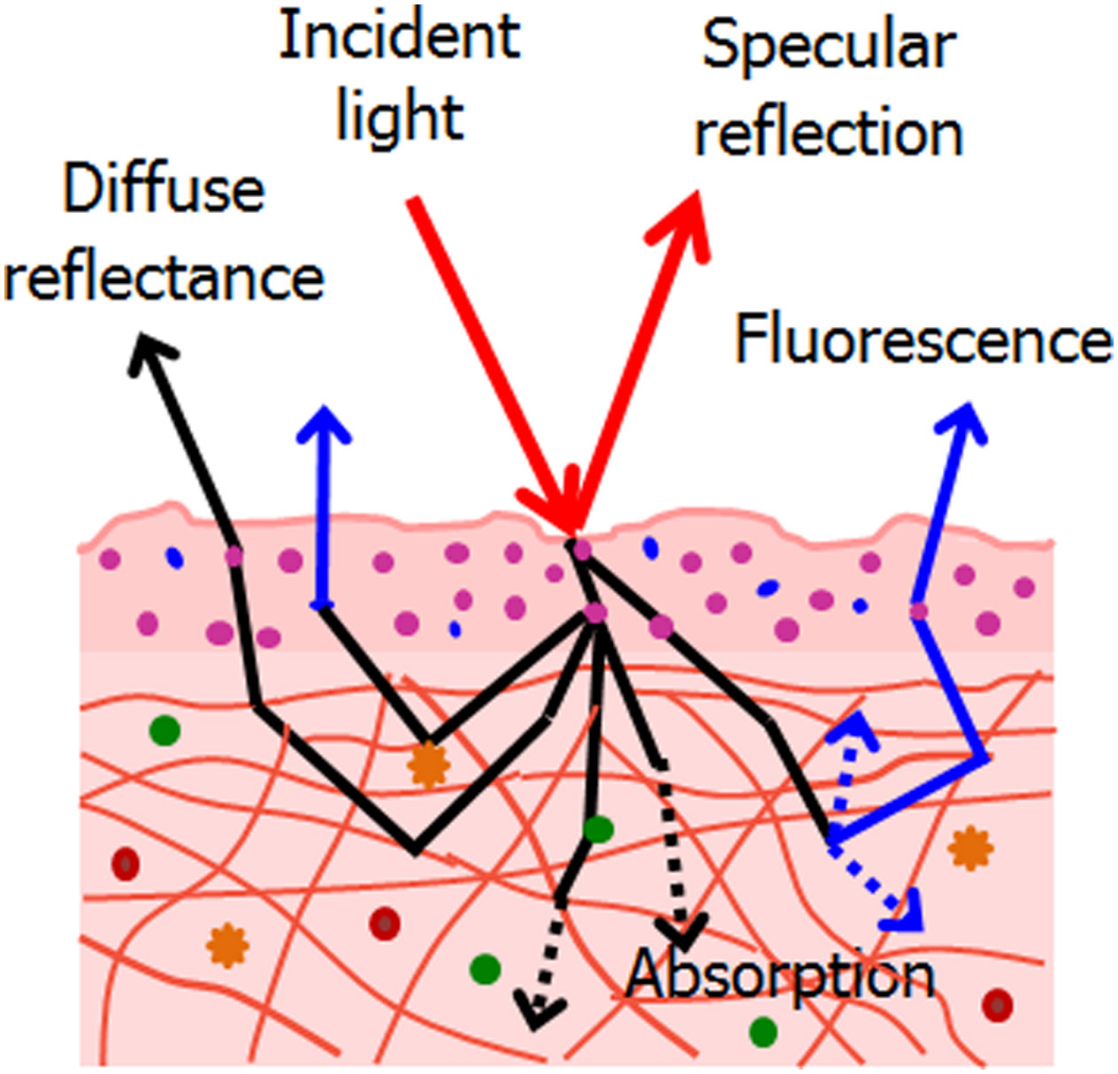
Cross-section view of biological tissue: epithelial layer (top) and adjacent connective tissue. Basic mechanisms of the interaction of polarized light with biological tissue include: (i) specular reflection from the air-tissue interface. In the case of surface roughness, one measures the superposition of different secularly reflected beams; (ii) diffuse reflectance related to the elastic scattering of light refracted through the air-tissue interface; (iii) light absorption within tissue; and (iv) auto-fluorescence—absorbed light can be re-emitted at longer wavelengths.

Light interacts with biological tissue through various mechanisms, including absorption, scattering, reflection/refraction, and fluorescence, as schematically presented in Fig. 8. The optical signal detected on the surface of biological tissue results from the interaction of the probing light with internal layers and macroscopic structural inclusions.

In a particular biomedical application, for precise analysis of the measured optical signals, it is essential to comprehend the contribution of each mechanism and to differentiate between bulk and bio-surfaces effects. The application of polarized light^283^^,^^284^ has proved its potential for addressing the latter problem (so-called, polarization gating). Generally, polarized light–biological tissue interaction analysis requires modeling approaches. The most common polarized Monte Carlo algorithms solve the vector radiative transfer equation (VRTE) and calculate the 3D spatial distributions of both intensity and polarization of light.^285^^–^^288^ In this approach, we use the intuitively simple description of light as a flux of photons and can model the real 3D geometry of the experimental system. However, most open-source polarized Monte Carlo codes focus primarily on modeling multi-layered stacks of turbid tissue-like scattering medium with parallel plane interfaces and monodisperse scatterers. The majority of the state-of-the-art Monte Carlo codes do not take into account tissue heterogeneity, optical anisotropy, and/or the complex topography of tissue surfaces.

In the following, we outline the forthcoming advancements in the polarized Monte Carlo approach, which will facilitate comprehensive and accurate modeling of polarized light interaction with biological tissues, considering the influence of bio-surfaces.

### 11.1 Complex Geometry of Actual Tissue

A polarized Monte Carlo model capable of simulating multilayered stacks with non-planar interfaces that account for the complex geometry of actual tissue^288^^–^^290^ will be a step toward optical biopsy of tissue because it will provide an estimation of the absorption and scattering properties of the epithelium (topmost layer of tissue where the majority of cancers start) concerning those of the underlying tissue.

### 11.2 Machine Learning

Linking machine learning and deep learning with the Monte Carlo modeling of polarized light propagation in turbid tissue-like scattering media offers a wealth of opportunities for advancing quantitative tissue imaging, definitive diagnosis, and biomedical robotic systems development. It can lead to more accurate and efficient simulations, enable advanced image reconstruction techniques, and facilitate personalized medicine approaches in the field of biophotonics and biomedical optics.

### 11.3 Poly-Disperse Anisotropic Scattering

By incorporating poly-disperse anisotropic scatters,^290^^–^^292^ a more realistic description of biological tissue can be achieved, accounting for the presence of nuclei cells of a few microns, sub-micron cell organelles, the extracellular matrix of collagen and elastin, as well as nerve fibers. The presence of fibers is the source of tissue birefringence (both intrinsic and of the form) and strongly affects the polarized signature of tissue.^293^^–^^295^ Although some groups have reported including infinite cylindrical scatterers in their Monte Carlo models,^296^ further comparative studies are necessary to validate this assumption.

### 11.4 Surface Roughness

There is evidence that polarimetric measurements of tissue surface roughness may bring complementary diagnostic information.^297^ The modeling of tissue surface roughness has been effectively tackled in the polarized Monte Carlo modeling approach.^298^ The studies demonstrated that the polarimetric signature of light scattered by a rough surface may be dominant compared with the bulk scattering. The open-source Scatmech code^299^ developed at NIST by T. Germer includes the models for diffuse surface scattering for small roughness values. Further, more comprehensive developments are required to incorporate significant tissue surface roughness, such as employing the facet model, and seamlessly integrate rough interfaces within the multi-layered tissue models.

### 11.5 Auto-Fluorescence

The Monte Carlo modeling of tissue auto-fluorescence involves considering the impact of tissue surface, the spatial distribution, and the fluorescent properties of specific tissue chromophores, as well as processing non-elastic scattering events.^300^^,^^301^ In fact, with further advancements, the combined analysis of re-emitted light polarization and optical sectioning is expected to enable the detection of any preferential orientation of tissue fluorophores at the epithelial layer. This insightful information holds potential as an additional optical marker for tissue diagnosis.

### 11.6 Modeling Acceleration

A Monte Carlo approach is parallelizable because each photon’s trajectory within biological tissue is calculated independently of other photons in the packet. The results of using GPU-based Monte Carlo software have already been reported by several groups.^302^^,^^303^ The adoption of this acceleration strategy will be beneficial for using polarized Monte Carlo modeling of optical experimental studies of biological tissue for a wide range of applications.

### 11.7 Coherent Effects

Major Monte Carlo codes are developed within the framework of the Stokes-Mueller formalism, which needs to provide information about the absolute phase. One needs to adapt the Jones formalism^304^ to model the coherence effects^305^^,^^306^ during light propagating within a scattering medium. Recent theoretical advancements in accounting for coherence effects offer the potential to reconcile both formalisms^307^^–^^309^ and effectively consider the vectorial nature of light while maintaining the computational efficiency of polarized light interaction with tissue.

### 11.8 Complex light with Orbital Angular Momentum

As computational power and modeling techniques continue to advance, the Monte Carlo modeling of shaped light propagation through tissue-like scattering media is likely to open new avenues for understanding light-matter interactions and drive innovation in various fields of science and technology. Monte Carlo modeling of shaped light carrying Orbital Angular Momentum (OAM) can aid in the development of advanced biomedical diagnostics. Through Monte Carlo evaluation of the optimal configuration of shaped light with OAM in the context of complex light interactions with bio-tissues, the potential arises to extract more precise information about the properties of bio-surfaces and tissue microstructure. Commencing with the emulation of Gaussian light propagation through the scattering medium^310^ and incorporating recent strides in modeling Laguerre-Gaussian beam propagation within turbid media, a realm of new possibilities unfolds. The prospects of Monte Carlo modeling of shaped light with OAM propagation through tissue-like scattering media are highly encouraging and encompass substantial potential across diverse realms of research and application.

### 11.9 Quantum Light

Extending Monte Carlo modeling of polarized light propagation in turbid tissue-like scattering media opens up new perspectives to explore the modeling of quantum light, e.g., such as entangled photons, interacting with biological tissues, which holds immense potential for advancing quantum-enhanced biomedical research, quantum sensing, and imaging. Quantum light interactions may offer advantages in sensing small changes in biological tissues, such as subtle structural variations or biomolecular processes. Monte Carlo modeling, in particular, can serve as a guiding force for the development of quantum-enhanced biosensors, surpassing classical limits in resolution and sensitivity. In addition, it holds implications in quantum optogenetics and quantum-enhanced neuroimaging, enabling non-invasive and high-resolution probing of neural activity. Furthermore, it opens up new possibilities in quantum entanglement spectroscopy, offering valuable insights into molecular structures and processes.

## 12 Optical Fibers Made of Bio-Inspired Materials for Biomedical Applications

**Section Author**: Patryk Sokołowski (Gdańsk University of Technology)

Biomaterials are widely used in biomedical applications, including sensing, imaging and delivery of light to tissue. The biomaterials include nontoxic, noncarcinogenic, nonthrombogenic, and nonimmunogenic materials, such as glasses, ceramics, some metals, and polymers.^311^ Only part of those materials fulfill the conditions for guiding light in the range of 1 mm to 1 m, which is of interest in most biomedical applications.^312^ Beyond conventional optical materials such as glass and polymers, the biocompatible and in addition biodegradable biomaterials are needed. Biodegradable fibers are not only to the advantage of the natural environment, but also to the benefit of an increasing number of new applications in biomedicine.^313^ The natural biomaterials such as silkworm and spider silk, agarose and alginate gel, gelatins, hydrogels, cellulose, and chitosan are used to produce optical fibers.^314^ Due unique properties of optical fibers such as small size, flexibility, immunity to electromagnetic interference, capability for direct optical measurements, high sensitivity, and the ability to design multiplexed or distributed sensing systems, fibers have found applications in biomedical imaging and sensing physical (temperature, pressure, strain) and biochemical (biomarkers, gases, pH) parameters.^315^^,^^316^ Biodegradable materials would be implanted in a patient’s body during the required treatment period, as well as left in the body after treatment and allowed to degrade over time without needing surgical removal.^317^

### 12.1 Recent and Future Challenges

Despite the promising possibilities of natural biomaterials for biomedical applications low level of attenuation is hard to achieve. Biomaterials have significant absorption below 350–400 nm, sometimes with an absorption peak in the visible spectrum. It can be a problem, because absorption is undesirable for light delivery. Most of the newly developed biomaterial fibers and waveguides have losses of a few dB/cm. It is relatively high compared to silica optical fibers with a loss coefficient of a few dB/km.^314^ Next challenge for biomaterials is optimizing biodegradation time and improving the stability of mechanical and optical performance. Because of biodegradation, the optical performance decreases with time after implantation.^318^

### 12.2 Advances in Science and Technology to Meet Challenges

Cellulose is one of the low-cost, high-water solubility, nontoxicity, biocompatibility, and biodegradable materials. Native cellulose RI is around 1.47; the regenerated cellulose usually has a value around 1.51.^319^ Modification of cellulose usually increases RI. Few fibers made of different cellulose materials and different cladding have been reported, as regenerated cellulose, acetate cellulose, and methylcellulose.^320^ Carboxymethyl cellulose (CMC) cladding-free fibers reported by were successfully applied in touch sensing and respiratory rate monitoring. In the article, fibers in diameters of 125−323  μm were fabricated and 280-μm mechanical and optical parameters have been reported. The fibers were found to be strong but not stretchable. The tested tensile strength ranged from 125 to 150 MPa. Attenuation constant values ranged from 1.6 to 2.7 dB/cm when measured at 637 nm, transmission window was found to be 550–1350 nm. The problem with cellulose fibers is disintegration when exposed to water. To avoid this problem, the carboxymethyl cellulose fibers were heat-treated to impart water resistance to them. Fibers were found to become water resistant upon the heat treatment and turned yellow. Yellowing of cellulose is attributed to the oxidative reactions, but does not affect major chemical changes.^321^

Another natural material to produce novel optical fibers is silk. Refractive index of fiber made from spider silk or silkworms was both reported as around 1.54.^322^ For now, spider silk is an improper material for fibers, because losses are estimated to be 8.8 dB/cm.^323^ Synthetic silk can be used to make fibers. Genetically engineered spider silk protein was used to produce a fiber with RI = 1.7, and the optical loss for the waveguide was 0.8 ± 0.1 dB/cm in air and 1.9 ± 0.3 dB/cm in mouse muscles.^324^ Spider silk fiber functionalization with metal for RI sensing was recorded.^325^

**Fig. 9:**
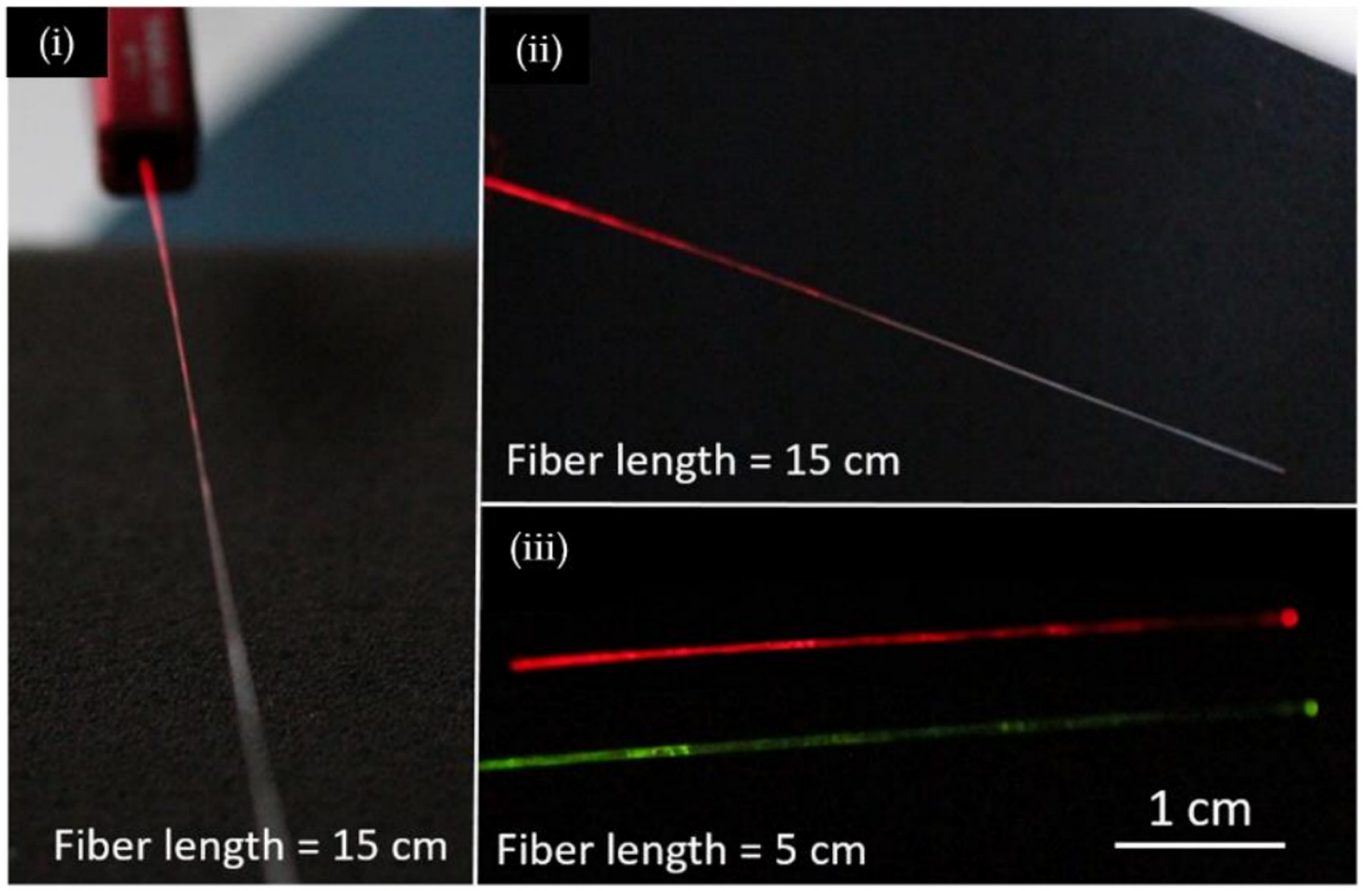
Optical fiber consisting of regenerated cellulose as core and recombinantly produced spider silk proteins eADF4(C16)-CBD as cladding material. Panels (i) and (ii) show a 15-cm long fiber segment at a wavelength of 670 nm. In panel (iii), 5-cm-long fiber segments are shown at a wavelength of 538 nm (green) and 670 nm (red). Reproduced with permission from Ref. 326.

The combination based on cellulose and spider silk allows for making a biodegradable optical fiber with attenuation of 1.91 dB/cm at 878 nm. For this purpose, the regenerated cellulose fibers are used as the core and recombinantly produced spider silk proteins as the cladding material (Fig. 9). For these fibers theoretical data rate was calculated to fulfill the performance of a modern 10 Gbps Ethernet cable at a distance of around 0.5–1 m.^326^ Due to the mentioned beneficial properties, the fiber could be surgically introduced into human or animal tissue for medical applications.

Recently, fiber optics from agar or agarose has also been developed. Fiber optics made of agar or agarose is fabricated by mould, so different shapes of core can be manufactured. Refractive index depends on agarose concentration. For 2% agarose concentration is around 1.33, but it can be changed with sucrose. Optical loss for 2% w/v agarose fibers was evaluated by the cut-back method using a HeNe laser, yielding 3.23 dB/cm.^327^ Because of high attenuation, only a few centimeters of fiber can be used in biomedical applications.

Hydrogels present remarkable characteristics in terms of tailoring optical and mechanical properties of the material by controlling the polymer water ratio, yielding transparency, flexibility, and stability.^327^ Hydrogel-based optical fibers are reported to have an excellent tensile strength of up to 2.27 MPa, the ability to stretch more than nine times their original length, and the ability to self-heal; biodegradability was not reported.^328^ Due to that, hydrogel optical fibers can remain functional for a period of time in living organisms. The promising biodegradable hydrogel is a PDLLA poly(d,l-lactic acid) material for solutions, where relatively fast degradation is required. For PDLLA fibers, degradation was measured at 84% molecular weight loss over a period of 3 months. Degradation time depends on the thickness, morphology of the polymer, and on the implantation site. Larger diameter degrade faster than those with smaller diameters. Optical parameters for PDLLA fibers are attenuation 0.16 dB/cm at 650 nm and 0.13 dB/cm at 850 nm, tensile strength was not reported.^329^

Finally, the development of new biomaterials is still ongoing. Already, optical fibers made of these materials can be used in biomedicine with satisfactory results. However, recent reports on new biomaterials focus on optical parameters and often do not report the tensile strength of the fibers or biodegradability. If the fiber should not be biodegradable, it is always possible to coat it with additional materials to slow down or prevent the biodegradation. Development of both optical and mechanical parameters is crucial. To further advance this field, it is crucial to develop fiber optics made with biomaterials and test with real biological samples. Biomaterials should be tested not only to deliver light to tissues but also as sensors.

## 13 Hyperspectral Imaging for Biomedical Applications

**Section Authors**: Jošt Stergar (Jozef Stefan Institute and University of Ljubljana), Urban Simončič (Jozef Stefan Institute and University of Ljubljana), and Matija Milanič (Jozef Stefan Institute and University of Ljubljana)

Hyperspectral (HS) imaging is an emerging modality that complements the traditional methods for examining structural and chemical properties of biophotonic samples, ranging from photonic surfaces to biological samples using light-sample interaction. In effect, it combines the strengths of spectroscopy with the versatility of imaging. Due to the rich data acquired by hyperspectral (HS) imaging, which offers broad insights into both sample physical and chemical structure, the perspectives for application are diverse. The advantages of hyperspectral imaging, being a non-contact, noninvasive, and generally affordable methodology, are well suited for translation into multiple disciplines, including medicine and biology,^356^^,^^357^ remote sensing,^358^ food science,^359^ and cultural heritage.^360^ The inherent sensitivity of hyperspectral imaging to contrast sources below the imaging resolution presents an exciting avenue into multi-scale imaging. Here, we present a list of challenges that will drive the future directions of the research.

### 13.1 Current Challenges and Future Directions

The broader adoption of hyperspectral imaging hinges on overcoming multiple challenges, which must be addressed in the future and are presented in the continuation.

#### 13.1.1 Standardization

Many hyperspectral (HS) imaging systems^361^^–^^363^ were developed to complement commercially available ones in recent years. Although the individual systems perform admirably in spectral acquisition, harmonizing the systems is an essential next step to enable multicentric studies, thus facilitating comparison and validation of experimental data. The challenge, therefore, lies in the standardization of HS imaging, which can be achieved using different approaches. First, the development and characterization of systems and verification against reference modalities and other systems must be performed. This goal depends on further developing accessible, stable, and reliable tissue-mimicking phantoms that can be exchanged between laboratories.

#### 13.1.2 Data processing

Hyperspectral images are three-dimensional datasets comprising hundreds of images at different wavelengths, containing thousands upon thousands of pixels. In this respect, processing HS data is an especially computation-heavy task, and data processing algorithms that can handle such datasets in an acceptable time are needed. Data processing consists of (a) pre-processing (spectral normalization, curvature, and height normalization, illumination inhomogeneity compensation, noise reduction, feature extraction, segmentation, etc.), (b) processing (optical properties extraction, tissue/sample properties extraction, classification/detection, etc.), and (c) post-processing (pseudo-image presentation of the properties and classifications, calculation of specially designed indices, predictions). Despite the wealth of algorithms available for all the processing steps, many open challenges exist. First, most algorithms consider spectral data decoupled from the spatial information, so research into spatial-spectral algorithms is needed. Second, the processing speeds present a unique challenge due to the sheer amount of data. Simple models, such as the Beer-Lambert law,^364^ are fast but are usually limited in their usability by strict restrictions on their validity. More advanced approaches might include Monte Carlo simulations, which are accurate but time-consuming. Novel algorithms are required to achieve near real-time processing speeds, with machine/deep learning as one possible solution. The final challenge lies in the sometimes severely ill-posed nature of the sample properties extraction problem, where multiple local minima exist. This challenge could be solved by algorithms integrating verification of the results in the processing pipeline with actions to remedy the mistakes stemming from the ill-posed nature of the problem.

#### 13.1.3 Equipment development

Traditionally, HS imaging systems are based on one of the traditional approaches, such as filtered illumination, push broom (spatial scanning in combination with an imaging spectrograph), or filtered detection.^365^ These approaches are tried and tested, but can be too slow for some applications that observe sample dynamics. An exciting direction to improve acquisition speeds is snapshot detectors that acquire the whole hyperspectral image in a single exposure, with mosaic cameras becoming more popular. In contrast to other HS imaging methods, they are small, light, and relatively inexpensive. They provide video-rate spectral imaging, which enables observation of transients, reduces motion artifacts, and can be used for surgical guidance. The challenges, however, lie in the spectral decomposition of limited bands defined by spectral peaks in the Fabry-Perot filters, developing reliable demosaicing algorithms, achieving adequate illumination, and integrating telecentric optics into imaging systems. Finally, a fixed set of spectral bands in the commercially available mosaic cameras presents a unique challenge in optimizing processing algorithms.

#### 13.1.4 HSI-derived metrics and datasets

Hyperspectral imaging provides a plethora of structural and biochemical information through the spectral signatures of tissue constituents. The volume of information within images acquired with a calibrated system is a truthful representation of the actual imaged object. It can thus enable the development of novel HSI-based metrics. One possible application of this “optical twin” representation is the development of simple HS imaging-derived matrices such as tissue indices. Although the idea is quite old, the complete spectral information within the image, combined with more thorough approaches for spectral analysis, provides a unique opportunity to develop such metrics. Due to the relative speed of calculation and the possibility of real-time processing, the tissue indices are expected to reappear as rapid, clinically relevant data indicators in studies, including hyperspectral imaging. The challenge lies in developing and verifying such indices. The scarcity of publicly available hyperspectral images in biological tissues and biosimilar materials makes developing such metrics even more challenging. Further development of HS imaging-derived metrics that focus on essential features and thus reduce image size for storage and sharing hinges on expanding available datasets for HS imaging analysis algorithm development.

### 13.2 Advances in Science and Technology to Meet Challenges

Addressing the prerequisite for standardization, the development of tissue-mimicking phantoms that can be used for standardization is lively, with polymer matrix phantoms at the forefront.^366^^–^^368^ These phantoms provide the stability needed to address the harmonization challenge of hyperspectral imaging but need further development into predictability and repeatability.

The pre-processing and noise reduction have been successfully addressed using algorithms such as MNF and moving averages. Simple algorithms enable the detection of spectrally distinct areas, a hallmark example being PCA. Addressing the challenge of rapid spectral data processing, possible candidates for algorithms that could see more use in the future are machine/deep learning algorithms (RS, SVM, NN, deep networks)^369^ that transfer the computation burden to the training stage, analytical solutions of the radiative transfer equation^370^ that provide solutions almost instantly in simple geometries or an inverse adding-doubling algorithm^371^ that, when implemented on a graphical processing unit, offers rapid solutions to the direct problem once the solver is initiated.^372^ Further development of the presented methods and possible additions to the repertoire will improve processing times, bringing spectral processing closer to real-time analysis.

Different approaches have been used to address the challenge of image speed acquisition to date. Image mapping spectrometers, borrowed from astronomy, show great promise, but the resolution can be limited.^373^^–^^375^ Alternatively, mosaic or spectrally resolved detector array (SRDA) cameras readily emerge as a new alternative to classical hyperspectral imaging.^375^ Due to their fast acquisition rates and sufficient resolution, they could acquire images appropriate for surgical image guidance using PCA for spectral analysis.^376^ Advanced approaches employing mosaic cameras enabled promising methods such as extended depth of field imaging.^377^

In many recent studies, HSI has shown great promise in introducing novel metrics, ranging from the evaluation of inflammatory conditions to the monitoring of murine tumour models in biomedical applications,^364^^,^^378^^,^^379^ through improved cinematographic film restoration with the help of machine learning,^380^ to applications in food science.^359^^,^^381^ In all these fields, hyperspectral imaging-derived metrics facilitate diverse aims, from detecting and grading diseases, restoring cinematographic films, to detecting contaminants in food.

## 14 Terahertz Sensing and Imaging of Biological Surfaces

**Section Authors**: Nikola Vuković (University of Belgrade), Aleksandar Demić (University of Leeds), Dragan Indjin (University of Leeds), and Jelena Radovanović (University of Belgrade)

Terahertz (THz) photonic technology has a groundbreaking potential for application to sensing and imaging of biological surfaces. This interdisciplinary area has evolved in the past two decades offering state-of-the-art sensing systems from which biological imaging could greatly benefit owing to the unique spectrum of biomolecules. Biomedical applications span from hydration sensing in leaves to skin cancer diagnosis using THz *in vivo* imaging.^382^^–^^388^ High sensitivity to water, resonance with biomolecules, favorable spatial resolution, capacity to probe the water–biomolecule interactions, and nonionizing photon energy are some of the unique features of THz waves. Although a lot of effort has already been invested into developing THz experimental equipment, theory, and data analysis, it is not straightforward to make comparisons between the published works because of the lack of measurement protocols and instrumentation standards, and there have been some discussions recently concerning the biological safety of intense THz radiation.^382^

### 14.1 Advances in Science and Technology to Meet Challenges

Considerable progress has been made toward narrowing the “THz gap,” which symbolizes the deficiency of efficient THz emission and detection devices. Traditionally, THz systems used for sensing and imaging of biological surfaces could be classified as THz time-domain spectroscopy (THz-TDS) systems and continuous-wave (CW) THz systems. Both platforms have their advantages, shortfalls, and scenarios of applications and differ greatly in available sources/detectors, experimental protocols, and procured information. An advantage of THz-TDS imaging is the fact that exposure of the object is achieved with the use of a broad frequency spectrum, a picosecond-level time resolution, and a field-detection ability comprising amplitude and phase for the spectrum.^389^^–^^391^ Nevertheless, shortfalls are the expensive femtosecond laser and the slow image acquisition time.^392^ Continuous wave THz systems require a generator of CW THz waves, and possible candidates include backward-wave oscillator (BWO), Gunn diode, gas laser, quantum cascade laser (QCL),^393^ THz parametric source, and THz photomixer, which all differ greatly in principles of operation and features.^382^

QCLs are quantum solid-state devices utilizing the intersubband transitions in the conduction band.^394^ The performance of QCLs has improved significantly over the last two decades in terms of working frequency, operating temperature, and output power.^395^^,^^396^ THz QCLs are a single source of coherent radiation with milliwatt power in the frequency range of 1.2 to 5.4 THz and are generally divided into direct and inversionless lasers. The latter have operation based on the difference-frequency generation effect, and their emission can be tuned over a fairly wide frequency range of 1.4 to 5.9 THz.^392^ Although the inversionless QCLs operate at room temperature, they have a low output power of ∼0.2  mW, which significantly reduces their application potential.^392^^,^^395^ In comparison, direct THz QCLs with a Fabry–Perot resonator typically work at cryogenic temperatures, have a fixed frequency and output power up to ∼100  mW^397^ with the record achieved optical power larger than 1 W.^398^ Recently, novel design schemes have introduced a paradigm shift in designing LO-phonon structures with transitions farther from the resonant energy, which has enabled high-temperature performance >250 K in pulsed operation.^399^^,^^400^ Room-temperature operation of THz QCLs will be mandatory for mass adoption of this technology since it would significantly reduce the size and cost of the solutions. The advances in the technology of THz QCLs drive the necessity for novel light-emitting schemes,^401^^,^^402^ theoretical descriptions, comprehensive numerical simulations of the underlying physical processes,^397^^–^^404^ especially those related to MBE growth that may have a prominent impact on THz QCL transport: background doping, interface composition diffusion, and layer thickness fluctuations.^405^

**Fig. 10:**
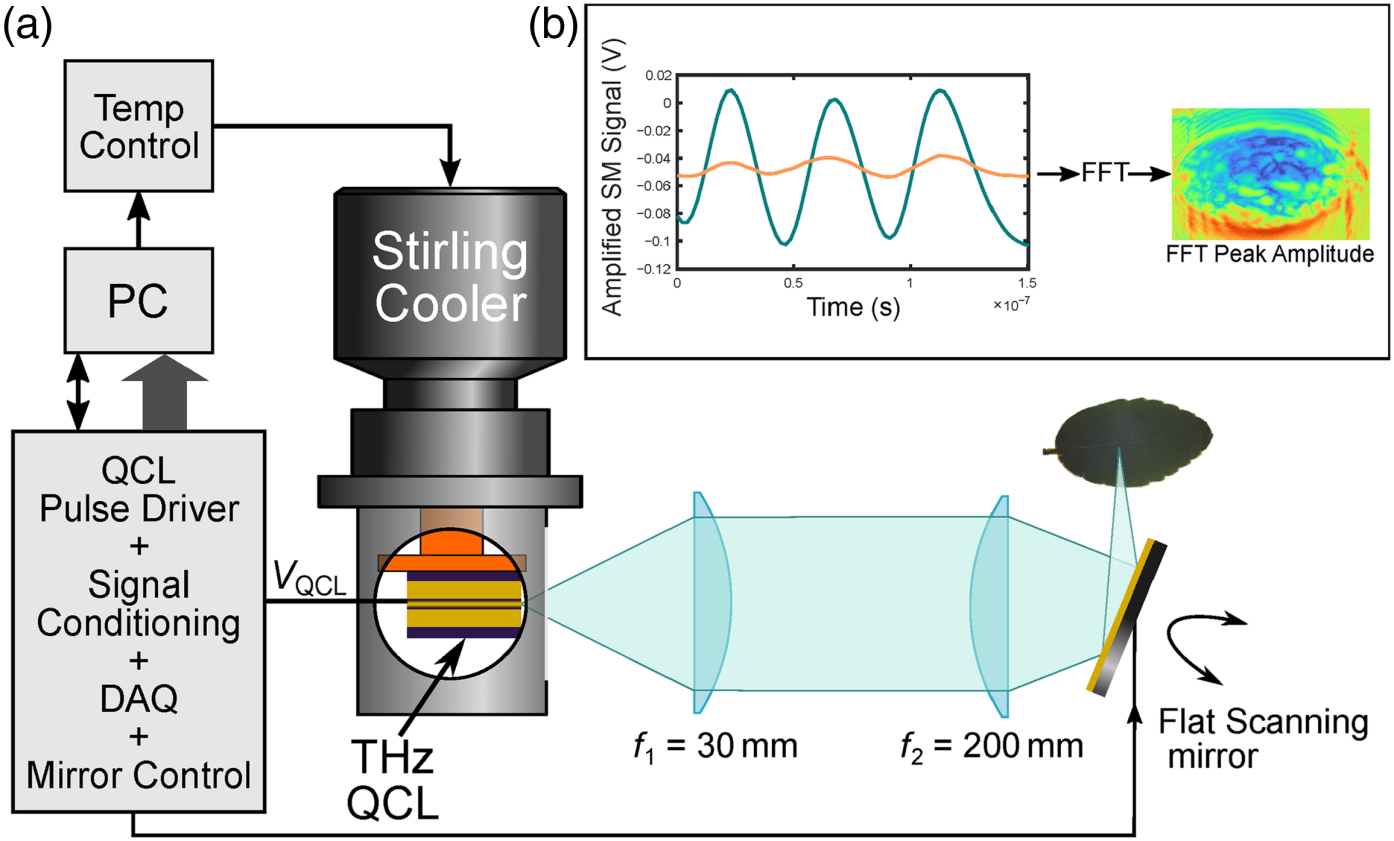
(a) Schematic of THz QCL LFI imaging setup showing fast scanning imaging mode. (b) Processing steps of the LFI interferogram (amplified self-mixing (SM) signal) into image pixels. Blue trace shows signal off bare metal backing. The orange trace shows a representative signal from the leaf. Reproduced from Ref. 406 under CC-BY license.

**Fig. 11:**
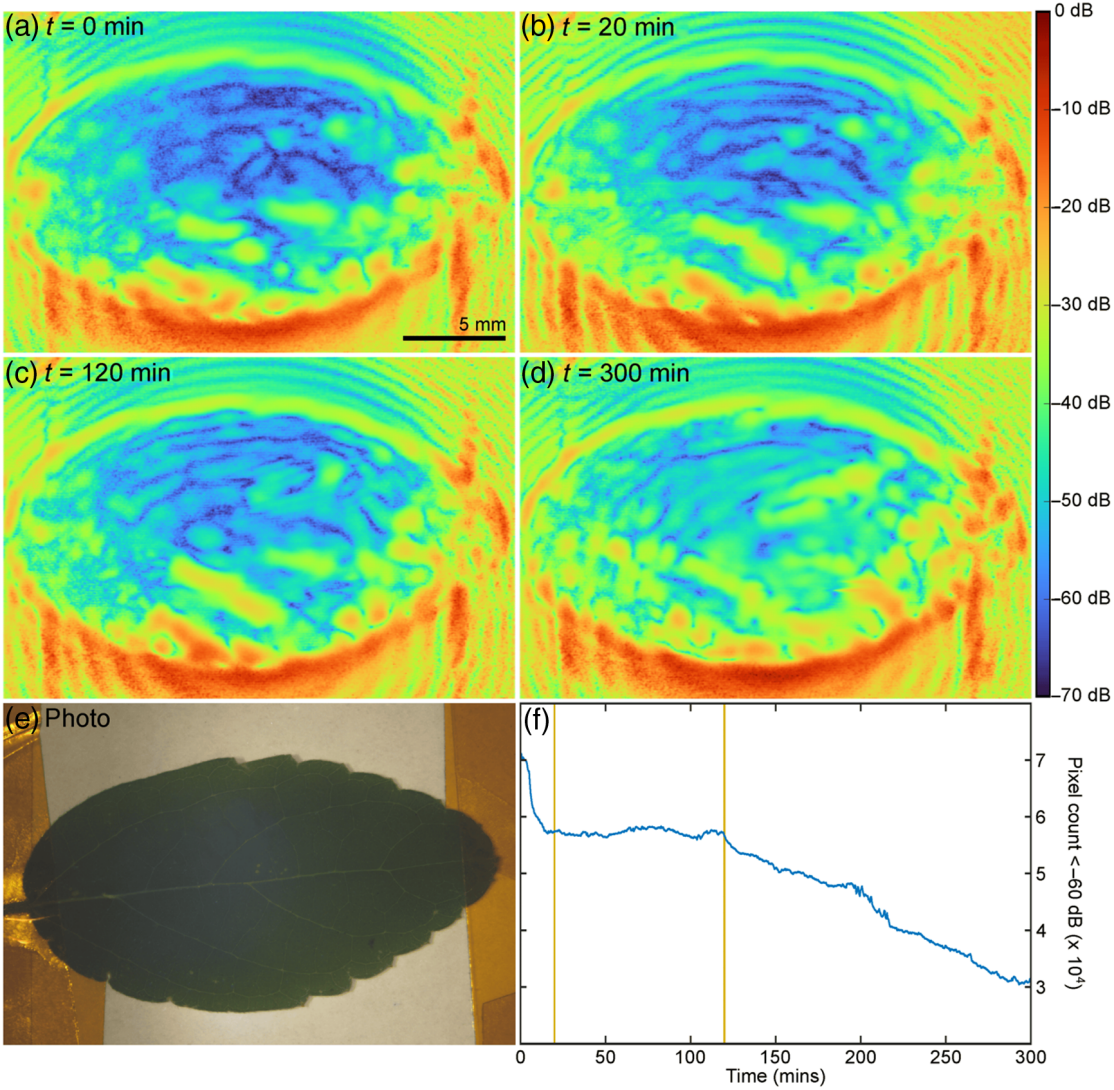
(a–d) LFI images of Celtis sinensis at t=0, 20, 130, and 300 min. (e) Photo of leaf after LFI scan (300 min +). (f) Change in count of “blue” pixels (Amp. < 60 dB) versus time. Horizontal lines indicate 20 and 120 min. Reproduced from Ref. 406 under CC-BY license.

A recent biosurface sensing application study was conducted through the use of THz imaging for hydration sensing in leaves,^406^^,^^407^ where researchers employed two complementary THz imaging techniques, THz-TDS and QCL laser feedback interferometry (QCL LFI),^408^ to make hydration maps of liquid water in the plucked leaves of Bambusa vulgaris and Celtis sinensis. Schematic of THz QCL LFI imaging setup and processing steps of the LFI interferogram into image pixels are shown in Fig. 10 (reproduced from Ref. 406). From the hydration maps, they gained useful information on spatial variations within the samples as well as hydration dynamics on various time scales. Spectral and phase information were retrieved using THz-TDS, showing dehydration effects on leaves, whereas THz QCL-based laser feedback interferometry proved useful for imaging fast dynamic variations in dehydration patterns shown in Fig. 11 (reproduced from Ref. 406).

Improving the sensitivity of biosensors by inducing larger changes in the THz response with only trace amounts of samples is challenging due to the long wavelength of THz waves compared with the sample thickness.^382^ Some of the most widely applied techniques to overcome this issue include metamaterials (MMs), waveguides, and THz surface plasmon polaritons. Metamaterials represent artificial media typically consisting of metallic subwavelength periodic structures that have tailored effective electromagnetic properties,^409^ and they have attracted a lot of interest in the past decade for application in THz technology Excited plasmonic modes in the metallic structures of the MM resonate at some particular frequencies and any small change in the dielectric environment caused by the trace amounts of analyte will introduce a measurable variation in the resonant frequencies.^405^^–^^428^ Theoretical studies and numerical simulations of physical properties in semiconductor nanostructures for metamaterial applications in the THz domain have also been conducted.^429^^–^^434^

Planar MMs are more commonly used for sensing applications. Their use in non-biomedical fields has disclosed extraordinary features that may be applied in the future for bioimaging and biosensing areas as well.^435^ Recent advances in metasurfaces for various bio-applications include optical chiral imaging,^436^ endoscopic optical coherence tomography (OCT), fluorescent imaging,^437^ super-resolution imaging,^438^ magnetic resonance imaging (MRI),^439^ and quantitative phase imaging (QPI). Applications of metasurfaces in biosensing include the detection of antibodies and proteins,^440^ DNAs,^441^ cells,^442^ and cancer biomarkers.^443^ Future directions envision potential translation of various metasurface functions, demonstrated for non-biomedical applications, into biomedical areas: metasurface-enabled adaptive optics for aberration correction and deep-tissue imaging,^435^^,^^436^ metasurface-enabled optical fibers for *in vivo* bioimaging and remote biosensing^437^^,^^438^ metasurface-based structured light beams for bioimaging,^439^^–^^445^ conformal metasurfaces for smart wearables and implantable health-care products such as optical bioimagers and biosensors,^446^ and metasurface-based optical tweezers for analysis of cells and bacteria.^447^

Certain animals with evolutionary advantages have inspired the development of many photonic biomimetic metasurface devices.^435^ Namely, the ability of certain small animals to sense the direction of sounds from coherent coupling of soundwaves between the ears has inspired a metasurface-based ultrasensitive photodetector.^448^ Desert ants were an inspiration for biomimetic metasurface–based coating for the passive effective cooling of objects.^449^ Polarization sensing of the compound eyes of mantis shrimps gave an idea for a design of the metasurface-based polarization filter device operating in NIR.^450^ The depth perception mechanism of jumping spiders stimulated scientists to develop a metasurface-based depth sensor.^451^ Mimicking the neural algorithms using photonics, observed in some special animals and plants, represents one of the natural solutions to the critical challenges in modern technologies. Jamming avoidance response in a weakly electric fish Eigenmannia is an example of such a natural neural algorithm, which has motivated scientists to solve a problem in wireless microwave systems.^452^ Similar concepts of bioinspired neural algorithms applied to THz technology would significantly enhance the existing progress in the field.

Intrigued by biological multicontrollability, authors in Ref. 453 have suggested the concept of bioinspired multicontrollable metasurfaces for THz applications, with numerous possibilities for design architectures. These devices would comprise electrically small elements made of diverse pixels, each of which is variously controlled by external magnetic, optical, electric, thermal, and mixed stimuli that produce a THz frequency response.

Another interesting direction for future THz optical devices, such as THz polarizers and waveplates, is the new hybrid materials, such as biomimetic aerogels based on cellulose nanofibers doped with various conductive materials, carbon nanotubes, silver nanowires, and MXenes.^454^ As an illustration, low refractive index and high birefringence and absorption have been demonstrated with the differently doped cellulose samples.^455^

In conclusion, although the THz technology development is essential for further improvements in sensing and imaging of biological surfaces, conversely, flora and fauna could become an excellent inspiration for evolution in the THz sensing and imaging field. There is still a lot of space for improvements in equipment, protocols and standards, theoretical analysis, and so on. Overall, future directions entail employing multidisciplinary research efforts to transfer existing approaches and technologies to biomedical applications as well as to devise novel ones.

## 15 Conclusion

This article presents complementary directions of research inspired by natural structures and phenomena for the development of photonic systems. It provides an overview of advances in the field, spanning nanoscale light–matter interactions, bioinspired photonic surfaces, and the creation of diagnostic tools and sensors. Despite numerous discoveries in biophotonics, major challenges remain unresolved, including label-free biomolecular specificity, quantitative imaging and sensing methods, and single-molecule detection. Progress is further constrained by the demand for low-cost, lightweight, and miniaturized devices that preserve essential optical, mechanical, and electronic properties.

To address these challenges, this roadmap reviewed multiple domains: light scattering by biological surfaces for biomedical applications such as structural characterization, disease diagnosis, and drug delivery; the genetic and structural mechanisms underlying biological photonic surfaces; and the development of scalable, biocompatible, and biodegradable photonic materials. Particular emphasis was placed on chiral nanomaterials, silica frustules, and artificial biomimetic surfaces inspired by butterflies, beetles, and other natural architectures. Complementary diagnostic techniques—including omics approaches, hyperspectral imaging, optical spectroscopies, and terahertz sensing—were also discussed, along with bioinspired phantoms for medicine and environmental monitoring that enable experimental validation and calibration of analytical systems. Simulations of light interactions with tissues were highlighted as a valuable tool for understanding bio-surface effects and advancing simulation-based design.

Together, these directions underline several unifying themes in the field: surfaces and scattering as fundamental mechanisms, bioinspired materials as a pathway to sustainable technologies, and sensing and imaging as the drivers of translation into biomedical applications. Overall, this roadmap provides a consolidated perspective on the emerging role of bioinspired materials and diagnostic systems in biophotonics, emphasizing the need to simplify system complexity, reduce device size, and ensure functionality for effective biomedical implementation.

## Data Availability

The data supporting the findings of this roadmap article are derived from previously published studies cited throughout the text. No new datasets or custom software were generated as part of this work. Additional details and data can be obtained from the corresponding authors of the original publications where applicable.
